# Disease flares with baricitinib dose reductions and development of flare criteria in patients with CANDLE/PRAAS

**DOI:** 10.1136/ard-2023-225463

**Published:** 2024-04-23

**Authors:** Kader Cetin Gedik, Ana M Ortega-Villa, Grace Materne, Andre Rastegar, Gina A Montealegre Sanchez, Adam Reinhardt, Paul A Brogan, Yackov Berkun, Sara Murias, Maria Robles, Susanne Schalm, Adriana A de Jesus, Raphaela Goldbach-Mansky

**Affiliations:** 1 Translational Autoinflammatory Diseases Section, LCIM, NIAID, National Institutes of Health, Bethesda, Maryland, USA; 2 Division of Pediatric Rheumatology, Children's Hospital of Pittsburgh of University of Pittsburgh Medical Center, Pittsburgh, Pennsylvania, USA; 3 Biostatistics Research Branch, Division of Clinical Research, NIAID, National Institutes of Health, Bethesda, Maryland, USA; 4 Division of Clinical Research, NIAID, National Institutes of Health, Bethesda, Maryland, USA; 5 Boys Town National Research Hospital, Omaha, Nebraska, USA; 6 University College London Great Ormond Street Institute of Child Health, London, UK; 7 Great Ormond Street Hospital for Children NHS Foundation Trust, London, UK; 8 Department of Pediatrics, Hadassah Hebrew University Medical Center, Jerusalem, Israel; 9 Hospital Universitario La Paz, Madrid, Spain; 10 Eskenazi Health Center, Indianapolis, Indiana, USA; 11 Rheumatologie im Zentrum, Munich, Germany

**Keywords:** Immune System Diseases, Inflammation, Outcome Assessment, Health Care

## Abstract

**Objectives:**

Patients with chronic atypical neutrophilic dermatosis with lipodystrophy and elevated temperature/proteasome-associated autoinflammatory syndrome (CANDLE/PRAAS) respond to the janus kinase inhibitor 1/2 inhibition with baricitinib at exposures higher than in rheumatoid arthritis. Baricitinib dose reductions to minimise exposure triggered disease flares which we used to develop ‘flare criteria’.

**Methods:**

Of 10 patients with CANDLE/PRAAS treated with baricitinib in an open-label expanded-access programme, baricitinib doses were reduced 14 times in 9 patients between April 2014 and December 2019. Retrospective data analysis of daily diary scores and laboratory markers collected before and after the dose reductions were used to develop ‘clinical’ and ‘subclinical’ flare criteria. Disease flare rates were compared among patients with <25% and >25% dose reductions and during study visits when patients received recommended ‘optimized’ baricitinib doses (high-dose visits) versus lower than recommended baricitinib doses (low-dose visits) using two-sided χ^2^ tests.

**Results:**

In the 9/10 patients with CANDLE with dose reduction, 7/14 (50%) times the dose was reduced resulted in a disease flare. All four dose reductions of >25% triggered a disease flare (p <0.05). Assessment of clinical and laboratory changes during disease flares allowed the development of disease flare criteria that were assessed during visits when patients received high or low doses of baricitinib. Disease flare criteria were reached during 43.14% of low-dose visits compared with 12.75% of high-dose visits (p <0.0001). Addition of an interferon score as an additional flare criterion increased the sensitivity to detect disease flares.

**Conclusion:**

We observed disease flares and rebound inflammation with baricitinib dose reductions and proposed flare criteria that can assist in monitoring disease activity and in designing clinical studies in CANDLE/PRAAS.

**WHAT IS ALREADY KNOWN ON THIS TOPIC**

- Chronic atypical neutrophilic dermatosis with lipodystrophy and elevated temperature/proteasome-associated autoinflammatory syndrome (CANDLE/PRAAS) is a rare autoinflammatory interferonopathy.
- A chronically elevated peripheral blood interferon (IFN) signature is a hallmark of incompletely treated CANDLE/PRAAS. Patients with CANDLE/PRAAS respond to the janus kinase inhibitor 1/2 inhibition with baricitinib at exposures higher than in rheumatoid arthritis.
- Defining and validating disease outcomes and/or activity criteria in patients with ultrarare diseases remains an ongoing challenge.
- Validated outcomes in autoinflammatory diseases have only been defined for interleukin-1 mediated autoinflammatory diseases.

**WHAT THIS STUDY ADDS**

- We observed substantial rebound inflammation and disease flares with baricitinib dose reductions in patients with CANDLE/PRAAS that allowed us to develop disease flare criteria.
- Baricitinib dose reductions of >25% resulted in disease flares in all patients, and reductions of <25% resulted in disease flares in 29% of the patients.
- We developed (1) clinical flare criteria based on clinical and laboratory changes and (2) subclinical flare criteria based on laboratory changes only.
- Flare rates using the proposed flare criteria were significantly higher during visits when patients received low doses of baricitinib than during visits when patients received higher doses of baricitinib.
- The addition of the IFN score as flare criterion can be considered in patients who can normalise the IFN score.

**HOW THIS STUDY MIGHT AFFECT RESEARCH, PRACTICE OR POLICY**

- Our study highlights the need to monitor disease flares when baricitinib dose reductions are implemented and cautions against large reductions or drug discontinuation.
- Our study provides a model for establishing and validating flare criteria using systematically collected data and addresses challenges faced when establishing outcomes in ultrarare inflammatory diseases.
- Disease flare criteria aid in monitoring treatment responses and in designing clinical trials in patients with CANDLE/ PRAAS in the future.

## Introduction

Chronic atypical neutrophilic dermatosis with lipodystrophy and elevated temperature/proteasome-associated autoinflammatory syndrome (CANDLE/PRAAS) is a rare autoinflammatory interferonopathy that is caused by loss-of-function mutations in genes that affect the 20S proteasome assembly and function. Patients respond to treatment with janus kinase inhibitors (JAKi), that reduce interferon (IFN) signalling associated with clinical improvement.^1–5^ In an expanded access programme (NCT01724580), 50% of patients with CANDLE/PRAAS achieved lasting clinical remission on baricitinib with normalisation of the IFN response gene signature^1^ but required higher exposure for optimal treatment compared with patients with rheumatoid arthritis.^3^ The development of BK viral reactivation in urine, particularly the development of BK viraemia and BK nephropathy, as well as cytopenias observed at higher exposure required for treatment of CANDLE/PRAAS have led to close monitoring of BK viral load and attempts to minimise drug exposure in patients with controlled disease, with the goal to reduce baricitinib exposure/doses to the lowest dose needed to preserve disease control.

The development of postdose reduction disease flares presenting with fevers, rashes, musculoskeletal (MSK) symptoms, headaches and elevation of inflammatory markers in patients undergoing dose reductions allowed us to leverage these data to develop the proposed flare criteria for CANDLE/PRAAS to assist in monitoring disease activity and in the development of clinical trials in randomised withdrawal studies in CANDLE/PRAAS in the future.

## Methods

### Patients

Patients with genetically confirmed CANDLE/PRAAS who received baricitinib in an open-label expanded access programme (NCT01724580) were included in this retrospective study.

### Assessment of clinical flares post baricitinib dose reductions

We performed a retrospective data analysis by screening the clinical database (INFORM) for baricitinib dose reductions that occurred during the study period from October 2011 through December 2020 (online supplemental table 1). Medical records and daily diary scores (DDS) were reviewed to assess worsening of clinical symptoms suggestive of postdose reduction disease flares.^6^ We extracted BK viral load in blood and urine before and after dose reductions. Clinical determination of disease flare was based on clinical judgement of the expert provider and included worsening of CANDLE/PRAAS associated clinical features (online supplemental table 2). We focused on acute changes in the context of a disease flare which do not include metabolic markers. The observed flare patterns in this study align with those previously reported in patients with active disease.^6 7^

10.1136/ard-2023-225463.supp1 Supplementary data

### Development of CANDLE/PRAAS disease flare criteria

Data were used from seven patients (P1, P3, P4, P5, P6, P7 and P10) to develop CANDLE/PRAAS disease flare criteria. The visit prior to dose reduction was used as reference visit to calculate postdose reduction changes. For the reference visit (see online supplemental table 3 for details), three patients (P4, P5 and P10) fulfilled remission criteria with ‘no disease symptoms (DDS <0.15), normal C reactive protein (CRP) and off glucocorticoids’ in accordance with the criteria established by Sanchez *et al*.^1^ One patient P3 was considered to have minimal disease activity (DDS <0.15), normal CRP and glucocorticoids of <0.15 mg/kg/day. Two patients (P1 and P6) had stable/controlled disease on glucocorticoids ~0.3 mg/kg/day and one patient (P7) required 0.8 mg/kg/day of prednisone and had baricitinib withheld due to high BK viral load in blood (the subsequent visits were therefore not used in the confirmation phase).

### Assessment of the CANDLE/PRAAS disease flare criteria in high-dose and low-dose baricitinib study visits

To assess the performance of the proposed flare criteria, we compared flare rates in six patients (P1, P3, P4, P5, P6 and P10) who were on low and high doses of baricitinib during the study. Four participants were excluded (P7: baricitinib was discontinued secondary to azotaemia; P2 and P9: did not have a low-dose period; and P8: did not have a dose reduction during the study period). Due to small sample size in this ultrarare disease, the same cohort was used for both development and assessment of the flare criteria.

Clinically ‘effective baricitinib doses’ were determined based on PK data (online supplemental table 4), that supported a currently recommended dosing regimen.^3^ Patient visits on lower than ‘effective doses’ were categorised as ‘low-dose’ visits and those on equal or higher than ‘effective doses’ were categorised as ‘high-dose’ visits.

To assess the performance of the proposed flare criteria, we compared disease flare rates during high-dose visits and low-dose visits (online supplemental materials).

### Statistical evaluation

Pairwise comparisons of DDS and laboratory biomarkers (reference vs flare visits) were performed via a two-sided Wilcoxon signed rank test. Time to flare was evaluated using a Cox proportional hazards model and data were displayed using Kaplan-Meier curves. The time to fulfilling the flare criteria was influenced by the timing of a postdose reduction blood draw. The maximal follow-up period consisted of 60 days. Flare rates were stratified by dose reductions of <25% and >25%. The proportion of disease flares was compared between the low-dose and the high-dose visits using a two-sided χ^2^ test of homogeneity. Logistic regression with generalised estimating equations was performed to determine the odds of flare in low-dose versus high-dose visits adjusting for prednisone dosing. No multiple comparison adjustments were performed. All analyses were done in R V.4.0.4. Statistical significance was considered if p <0.05.

## Results

### Patients with CANDLE/PRAAS who had baricitinib dose reductions developed dose-dependent disease flares

Baseline demographics and clinical characteristics of 10 patients with CANDLE/PRAAS were previously described (online supplemental table 5).^1^ The mean age at enrolment was 11.5 years (range 2.3–19.6).

**Figure 1:**
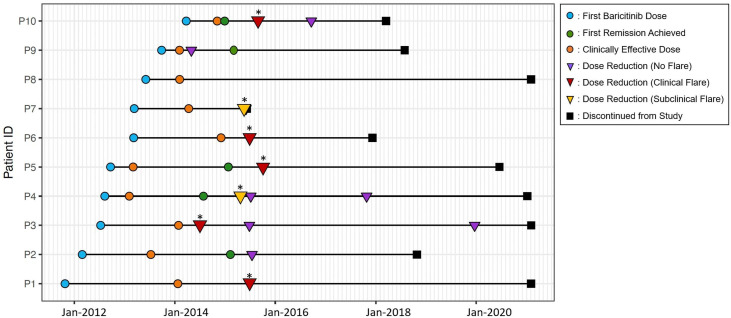
Timeline of baricitinib dose changes and development of chronic atypical neutrophilic dermatosis with lipodystrophy and elevated temperature/ proteasome-associated autoinflammatory syndrome (CANDLE/PRAAS) disease flare. Timeline of baricitinib initiation (blue circle), achieving the first remission (green circle), reaching clinically effective dose (orange circle), dose reduction not triggering a flare (purple triangle), dose reduction that triggered a clinical flare (red triangle), dose reduction that triggered a subclinical flare (yellow triangle) and study discontinuation (black square). Mean time to establish clinically effective dose was 1.06 years (±0.65 year) in 10 patients with CANDLE/PRAAS. Mean duration of baricitinib treatment was 6.3 years (±2.3 years). Of 10 patients, 9 had 14 baricitinib dose reductions. P8 did not have a dose reduction. Of 14 dose reductions, five (P1, P3, P5, P6 and P10) resulted in a clinical disease flare in five out of nine patients and two (P4 and P7) resulted in laboratory changes alone consistent with a disease flare with no clinical symptoms. *Indicates incidences of flare, clinical or subclinical.

During the study period (10/2011–12/2020), we identified 14 baricitinib dose reductions that occurred between April 2014 and December 2019 in 9 out of the 10 patients with CANDLE/PRAAS (figure 1). Following baricitinib dose reductions, we observed a concomitant decrease in BK viral load in blood and urine (online supplemental table 1); however, of the 14 dose reductions, 50% (7/14) resulted in flares. Five were associated with clinical symptoms and laboratory changes during the flare (P1, P3, P5, P6 and P10); and two flares were associated with laboratory changes (P4: CRP, absolute lymphocyte count (ALC) and white blood cell count (WBC) and P7: WBC, ALC and platelets (PLT)) without clinical symptoms. One patient (P7) with presumed BK nephropathy and elevated creatinine stopped baricitinib 8 days later. He developed macrophage activation syndrome (MAS), required treatment with pulse methylprednisolone and was ultimately treated with prednisolone 30 mg daily.

**Figure 2:**
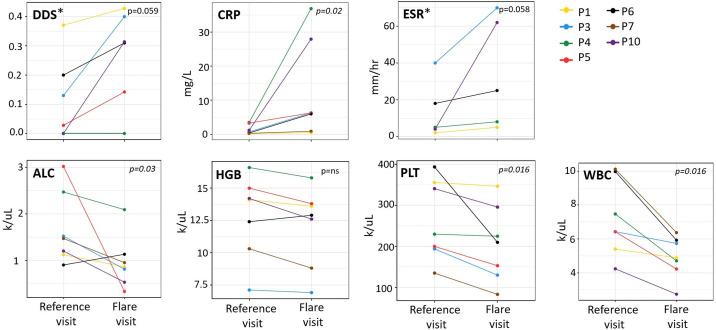
Acute clinical and laboratory biomarker changes with baricitinib dose reduction in seven patients judged to have a clinical or subclinical disease flare. This figure depicts comparison of the reference visit (=last visit before baricitinib dose reduction) with flare visit (=the first visit after baricitinib dose reduction) for DDS and the laboratory values for the patients who developed baricitinib dose reduction associated clinical and/or laboratory changes. Each parameter for each patient is graphed (see symbols used in upper right-hand corner of the graph for each patient). A two-sided non-parametric Wilcoxon signed rank test with uncorrected p values was used to underscore the descriptive representation. ALC, absolute lymphocyte count; CRP, C reactive protein; DDS, daily diary score; ESR, erythrocyte sedimentation rate; HGB, haemoglobin; PLT, platelets; WBC, white blood cell. *P7: DDS and ESR were not available for predose or postdose reduction visit or for both. P5: ESR was not available postdose reduction.

Flares were initially determined clinically based on the report of symptoms (including facial images of rash and periorbital oedema sent by email) that developed within days of the baricitinib dose reduction (not shown), physical examination (online supplemental table 2) and concomitant laboratory changes. We then assessed mean DDS changes before and after dose reductions (reference visit vs flare visit) to use a patient-reported measure to assess clinical disease activity. Available DDS (n=6) worsened between 15% to >500% (absolute change ranged between 0.055 and 0.314) (online supplemental table 2). Clinical flares postdose reduction were accompanied by changes in the six laboratory markers assessed, including CRP, erythrocyte sedimentation rate (ESR), WBC, haemoglobin (HGB), ALC and PLT (figure 2). One patient (P1) had an increase in the IFN score after dose reduction (online supplemental table 6a and online supplemental figure 1).

### Establishing CANDLE/PRAAS disease flare criteria

To establish flare criteria, we systematically assessed the seven flares that were clinically determined, five were associated with clinical and laboratory features, and two with only laboratory changes. The changes in the laboratory criteria comparing the reference visit (online supplemental table 3) with the flare visit included increases in acute phase reactants; CRP (91% to >500%), ESR (39% to >500%) and the presence of cytopenias; a WBC decrease between 34% and 41%, an ALC decrease between 15% and 89%, a PLT count decrease between 24% and 47% and a HGB decrease between 1% and 14% (online supplemental table 6a). Of the five patients with worsening of clinical symptoms, one patient (P1) had a change in only one laboratory marker (23% decrease in ALC from baseline), and four (P3, P5, P6 and P10) had changes in four laboratory markers. Two patients who had laboratory changes with no clinical symptoms had changes in three laboratory markers (P4: CRP, WBC ALC; P7: WBC, PLT, ALC) (online supplemental table 6a). We defined two types of disease flare criteria (table 1):

(1) Clinical flare criteria with clinical symptoms that were either observed on physical exam or photos or a DDS change by >15% plus at least two laboratory changes, and (2) subclinical flare criteria defined by the absence of clinical disease features and at least three laboratory changes.

**Table 1:** Definition of clinical and subclinical CANDLE/PRAAS disease flare

Definition	Laboratory biomarkers*
**Clinical flare criteria** A worsening in DDS† by a minimum of 15% or the documentation of flare symptoms in the medical record AND changes in two or more laboratory biomarkers	CRP (> 40% increase) ESR (> 20% increase) WBC (> 20% decrease) Platelets (> 20% decrease) ALC (> 15% decrease) HGB (> 15% decrease)
**Subclinical flare criteria** Less than 15% worsening in DDS† and/or no flare symptoms on physician evaluation AND changes in three or more laboratory biomarkers when compared with the reference visit	CRP (> 40% increase) ESR (> 20% increase) WBC (> 20% decrease) Platelets (> 20% decrease) ALC (> 15% decrease) HGB (> 15% decrease)

*Acute change in laboratory biomarkers with disease flares compared with the reference visit (last visit with documented laboratory biomarkers prior to baricitinib dose reduction period). For inclusion as flare criterion, the change of CRP and/or ESR score must result in a clinically abnormal value and an increase of greater or equal to 20% must occur.

†Mean DDS for 7 days (ranges from 3 days before and after the visit).

ALC, absolute lymphocyte count; CANDLE/PRAAS, chronic atypical neutrophilic dermatosis with lipodystrophy and elevated temperature/proteasome-associated autoinflammatory syndrome; CRP, C reactive protein; DDS, daily diary score; ESR, erythrocyte sedimentation rate; HGB, haemoglobin; WBC, white blood cell count.

**Figure 3:**
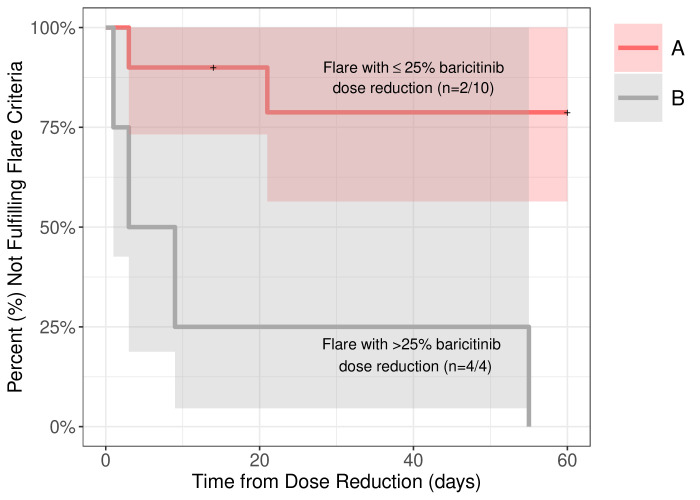
Time from baricitinib reduction to the fulfilment of chronic atypical neutrophilic dermatosis with lipodystrophy and elevated temperature/ proteasome-associated autoinflammatory syndrome (CANDLE/PRAAS) disease flare criteria (clinical and subclinical without interferon score). This Kaplan-Meier curve depicts the rate of disease flares (%) associated with baricitinib dose reductions in patients with CANDLE/PRAAS on y-axis, and time from dose reduction to flare in days on x-axis. Red line (group A) represents patient visits with a less than or equal to 25% baricitinib dose reduction (n=10) and the grey line (group B) represents occurrences of greater than 25% of baricitinib dose reductions (n=4). All four baricitinib dose reductions greater than 25% in group B resulted in a disease flare and fulfilled the flare criteria within 60** days. The proportion of flares in group A (20%) versus group B (100%) was significantly different (p=0.012). The time to flare in both groups was evaluated by Cox proportional hazards models and displayed using Kaplan-Meier curves. *P1 developed clinical symptoms with one laboratory abnormality postdose reduction and although he had a clinical flare, he did not fulfil the flare criteria and is not included in the count. **P7 developed symptoms several days after baricitinib dose reduction however did not have a blood draw until 55 days later when the disease was still active. P7 fulfilled the flare criteria on day 55 postdose reduction when the laboratory markers were available.

The flare criteria were then applied to all 14 dose reduction visits. Of the seven clinically determined flares, six fulfilled the flare criteria (four clinical flare visits (P3, P5, P6 and P10) and two subclinical flare visits (P4 and P7)); however, P1 did not fulfil the flare criteria. JAKi dose reductions that trigger flares depend on factors including the magnitude of the dose reduction, the actual baricitinib dose the patient received at the time of drug withdrawal and the level and duration of disease control at the time of dose reduction. In our patients, disease flares occurred in all four patients (100%) who had a dose reduction of >25%; two patients had clinical and two had subclinical flares. A dose reduction of <25% resulted in clinical flares in 2 (20%) of 10 dose reductions (figure 3 and online supplemental table 6a). We assessed P1 who had a clinically determined disease flare presenting with headaches, oral ulcers, MSK pain, fatigue but who did not fulfil the clinical disease flare criterion. He developed lymphopenia as the only laboratory flare criterion. However, P1 had IFN scores assessed before and after the dose reduction, and the 25-gene IFN score increased from 31.21 to 236.29 (cut-off 44.2). We therefore subsequently evaluated the inclusion of the IFN score as an additional flare biomarker in a subanalysis by only including visits that had an IFN score assessed. The time from baricitinib dose reduction to fulfilling the proposed flare criteria required a blood draw postdose reduction and ranged from 1 to 55 days postdose reduction (mean 15.3 ±20.7 days). One patient had clinical symptoms with DDS changes several days after dose reduction; however, this patient did not have bloodwork done until 55 days after the dose reduction. To include laboratory changes, the maximum follow-up period consisted of 60 days. We included this patient since: (1) the patient had clinical changes after baricitinib dose reduction; (b) she did not have another baricitinib dose adjustment until blood work was done; and (c) we considered her clinical and laboratory changes consistent with the clinical changes post baricitinib dose reduction.

### Confirmation of the CANDLE/PRAAS flare criteria

**Figure 4:**
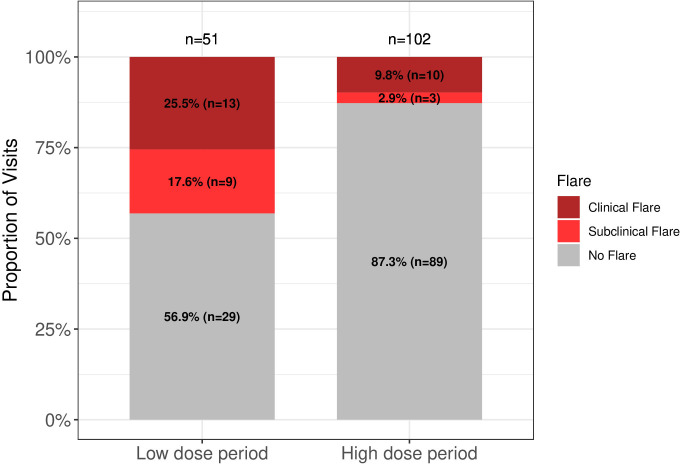
Proportion of disease flares comparing low-dose baricitinib visits to high-dose baricitinib visits. A total of 153 visits in 6 patients who had both high-dose (n=102) and low-dose (n=51) visits were identified and assessed. The proportion of visits that patients fulfil flare criteria during the low-dose period (43.14%) is significantly higher (p<0.0001) than during the high-dose period (12.75%). The proportion of visits that patients fulfil flare criteria during the low-dose and high-dose period was compared by using a two-sided χ^2^ test of homogeneity.

To further evaluate the disease flare criteria, we hypothesised that patients on optimised doses of baricitinib would fulfil disease flare criteria less often than patients on low baricitinib doses. A total of 153 visits in 6 patients (P1, P3, P4, P5, P6 and P10) who had both high-dose and low-dose visits were identified; of those, 102 occurred on an optimal dose baricitinib (high-dose visits), and 51 on lower than currently recommended/optimised baricitinib doses (low-dose visits). Disease flare criteria were fulfilled in 43.14% of low-dose visits compared with 12.75% of high-dose visits (p<0.0001) (figure 4). The median baricitinib dose during the high-dose period compared with low-dose period was 9.00 (IQR 2.00) mg/day versus 6.00 (IQR 3.00) mg/day respectively, p <0.0001. The median prednisone equivalent dose was significantly lower in the high-dose period compared with low-dose period, 0.00 (IQR 0.136) mg/kg/day and 0.149 (IQR 0.13) mg/kg/day, respectively, p<0.0001 (online supplemental figure 2). Adjusting for prednisone equivalent dose, higher odds of a flare in the low-dose period when compared with the high-dose period were found (p=0.03) (online supplemental figure 3). Lastly, the IFN score was assessed in 29/56 (52%) low-dose visits and 79/102 (77%) high-dose visits. The median IFN score was 254.46 (IQR 267.56) in the low-dose period, significantly higher compared with the high-dose period 45.93 (IQR 117.78) (p <0.0001) (online supplemental figure 4). Ranges of cut-off values of clinical and subclinical flare visits identified during the high-dose and low-dose visits and clinical scenarios that illustrate the use of the flare criteria are listed in the (online supplemental tables 6b and 7).

### Addition of IFN score criterion to the CANDLE/PRAAS disease flare criteria

The IFN score was assessed before and after dose reduction in 8 of 14 instances of dose reductions. It increased by >500% in P1 who had a clinical flare and remained within normal values in P4 who had a subclinical flare (online supplemental figure 1). Among six patients who did not fulfil flare criteria (seven visits), the IFN score increased by 233% in one patient (P1) (one visit), did not change or remained within normal limits in five patients (five visits). IFN score was not available in one visit (P4 second visit) (online supplemental table 6a).

In a subanalysis, we included the IFN score as biomarker criterion and determined the same cut-off of ≥20% that we used for ESR. Addition of the IFN score criterion to the proposed flare criteria increased the proportion of visits that patients fulfilled flare criteria (clinical and subclinical) from 37.93% to 58.62%, during the low-dose period and from 10.13% to 20.25% during the high-dose period; the difference remained significant (p<0.001) (online supplemental figure 5).

## Discussion

In this retrospective study, we observed substantial rebound inflammation and disease flares with baricitinib dose reduction. These findings allowed to develop flare criteria to assist with monitoring of disease activity, and to be used in designing clinical trials in CANDLE/PRAAS.

We observed more disease flares in patients with a larger dose reduction. In one patient, abrupt drug discontinuation triggered the development of MAS. Rebound flares with drug reductions and discontinuations are commonly seen in autoinflammatory diseases.^8–10^ Rebound flares are of particular concern when drugs with short half-lives are withdrawn, as they can trigger sudden, severe flares that can lead to organ damage. Serious withdrawal symptoms are well documented in patients withdrawing psychopharmaceutics that have short half-lives, including antidepressants and narcotics,^11 12^ but this is not generally considered in patients with systemic inflammation. In patients with interferonopathies, baricitinib dose adjustments may be necessary to reduce exposure and to limit baricitinib-related side effects, such as cytopenias and to reduce BK viral load in the blood to keep with safety standards developed for renal transplant recipients.^13–15^ In patients who achieve disease control on baricitinib, the lowest effective dose to maintain disease control should be sought.

Due to differences in pharmacokinetics in children and adults,^3^ which is caused by increased drug clearance and a higher volume of distribution in children, higher drug doses and shorter dosing intervals may be required in children to achieve similar exposure to adults. Dose decreases are therefore necessary as children grow and gain weight. Our data indicate that dose reductions should be implemented slowly and gradually and need to be carefully monitored.

Defining and validating disease outcomes and/or activity criteria in patients with ultrarare diseases remains an ongoing challenge.^16^ Validated outcomes in autoinflammatory diseases have so far only been defined for IL-1 mediated diseases and focus on the definition of disease remission^17 18^ or the reduction of a disease symptom score coupled with CRP.^9^ Instruments to assess disease symptoms include a DDS and/or a disease activity questionnaire such as the autoinflammatory diseases activity index (ADAI) that is assessed during disease attacks.^19 20^ Overall, these disease activity outcomes and flare descriptions align with accepted endpoints suggested for patients with juvenile idiopathic arthritis.^21^ We had defined remission for CANDLE/PRAAS,^1^ but disease flare criteria have not been developed. Our systematic analysis of patients’ flares by assessing paired visits before and after baricitinib dose reductions allowed extraction of parameters that changed during disease flares including concomitant increases in acute phase reactants, a drop in WBC, ALC and in PLT count. The degree of change in HGB was much smaller compared with the change observed in the other markers. We however kept HGB as a flare criterion, as we measured only acute changes, as in longer duration of suboptimal care, more pronounced HGB changes are usually seen.

A chronically elevated peripheral blood IFN signature is a hallmark of incompletely treated CANDLE/PRAAS. Prolonged normalisation of the IFN signature has so far only been seen in a subset of patients with CANDLE/PRAAS who carry autosomal recessive *PSMB8* mutations treated with JAKis including baricitinib.^1^ Our study suggests that addition of the IFN score criterion captures more disease flares that were associated with DDS increases and thus associated with clinical symptoms and increased the sensitivity of detecting a flare in particularly in lymphopenic patients who had low CRP. Inclusion of the IFN score as laboratory criterion identified six clinical flares that were not identified using the clinical flare criteria without the IFN score. Caveats when using the IFN score include substantial fluctuation in a given day^3^ when patients are not in remission. In active patients, a 20% variation in a single day does not flag a disease flare and variations should be interpreted with caution. The IFN score inclusion as criterion for disease flare is therefore only useful in patients with well-controlled disease who can normalise the IFN score. The proposed flare criteria without IFN score can be used in settings where the IFN score assessment is not routinely available. In clinical studies, inclusion of the IFN score to the proposed flare criteria would increase the number of flare visits detected particularly in patients with chronic cytopenias.

In this study, we assessed the disease criteria during periods when patients were treated with high-dose baricitinib, reflecting treatment with currently recommended doses,^3^ and during low-dose visits when patients received doses below those that are currently recommended. The criteria identified disease flares during these periods by comparing them to a reference visit that was established when patients were well controlled. Although the need for a reference can be considered a ‘limitation’, in practical terms it makes little sense to determine disease flares in patients with CANDLE/PRAAS who have chronically active disease. Comparing disease activity to periods when the disease is controlled allows an individualised assessment of flares and treatment adjustments and assists in longitudinal monitoring of disease activity. The criteria do not currently adjust for high glucocorticoid doses. However, when reference visits were established in our study, all patients were either off glucocorticoids and fulfilled remission criteria^1^ (P4, P5 and P10) or had more than 50% reduction (mg/kg/day) from baseline (P1, P3, P6 with P3 fulfilling remission criteria on low doses of glucocorticoids), except P7 whose glucocorticoid dose was maintained at a high dose to prevent disease flares in the context of baricitinib reduction and withdrawal due to the development of BK nephropathy.

The proposed flare criteria can serve as a valuable tool, empowering healthcare professionals to systematically monitor disease activity. The flare criteria may be particularly useful in assessing patients when lowering glucocorticoid or baricitinib dose and when evaluating the necessity for intensifying JAKi therapy in patients who develop postdose reduction flares. The criteria can also be used to make baricitinib dose adjustments during growth. The proposed flare criteria may become useful but require validation in other interferonopathies including Aicardi-Goutières syndrome (AGS) and stimulator of interferon genes (STING)-associated vasculopathy with onset in infancy (SAVI) when treatments become available that achieve similar disease control to JAKis in CANDLE/PRAAS. In addition to the value of these criteria in monitoring and objectively quantifying disease flares over time, the criteria can also be used to assess disease flares in clinical trials that randomise patients who achieved disease control to continued investigational drug use or placebo or standard of care control.^22^ Our flare criteria and assessment of flare rates during high-dose and low-dose visits facilitate powering clinical trials in patients with CANDLE/PRAAS but also caution that abrupt withdrawals can result in severe rebound such as MAS.

There are limitations to this study. The study was conducted retrospectively and did not have a prespecified control arm; the sample size is small. Further validation of the proposed flare criteria is warranted through testing in larger series and clinical trials. A strength is the prospective evaluation of patients for a median of 6.9 years which allowed the inclusion of 153 patient visits with complete laboratory evaluations. The criteria generated can also be used to generate data on disease flare rates in natural history studies in rare inflammatory diseases that can serve as comparison in the assessment of treatment interventions in rare diseases. Comparing treatment data to historical control data have been proposed as acceptable for regulatory approval of treatments for ultrarare diseases.^23^

In conclusion, our data should raise awareness of rebound inflammation during baricitinib dose reduction and alert caretakers to establish monitoring procedures in patients requiring dose adjustments. The ‘proposed disease flare criteria’ can aid in monitoring treatment response by assessing flare rates and in designing clinical trials in CANDLE/PRAAS in the future.

## Data Availability

Data are available upon reasonable request. Protocol (NCT01724580) and data can be made available upon request to the main author.
