# Supplementary material for: Disease flares with baricitinib dose reductions and development of flare criteria in patients with CANDLE/PRAAS
Source: Ann Rheum Dis. 2024 Apr 23;83(9):1181–8. doi: 10.1136/ard-2023-225463 (PMC11420725; doi:10.1136/ard-2023-225463)
Supplement: Supplementary data [file ard-2023-225463supp001.pdf]

SUPPLEMENTARY MATERIALS

Contents:

I. Supplementary Methods

1. **PATIENTS AND INCLUSION AND EXCLUSION CRITERIA**
2. **DAILY DIARY SCORE ASSESSMENT**
3. **DATA EXTRACTION**
4. **DEVELOPMENT OF CANDLE/PRAAS DISEASE FLARE CRITERIA**
5. **ASSESSMENT OF THE CANDLE/PRAAS DISEASE FLARE CRITERIA**

II. Supplementary Tables

1. **Supplementary table 1**: Baricitinib dose reductions and effects on BK viremia/viruria
2. **Supplementary Table 2** Summary of post-baricitinib dose reduction clinical symptoms and outcomes of clinical flare vs no clinical flare
3. **Supplementary Table 3** Summary of laboratory biomarkers, mean DDS, glucocorticoid data and clinical status at reference visits for patients Included in the confirmation of the flare criteria
4. **Supplementary Table 4** Summary of baricitinib dosing regimen at the time of baricitinib dose reductions
5. **Supplementary Table 5** Baseline demographics and clinical characteristics
6. **Supplementary Table 6a** Absolute values and percent changes of DDS and laboratory biomarker levels comparing the reference visit with the flare visit
7. **Supplementary table 6b**. Ranges of cut-off values of clinical and subclinical flare visits identified during the high and low dose visit phases are in range with the changes seen in the acute baricitinib withdrawals.
8. **Supplementary Table 7** Treatment decisions (glucocorticoid adjustments/baricitinib adjustments/no action) during “flare visits”

III. Supplementary Figures

1. **Supplementary Figure 1:** Acute Interferon (IFN score) change with baricitinib dose reduction

2. **Supplementary Figure 2:** Baricitinib and prednisone equivalent dose high baricitinib dose period versus low baricitinib dose period

3. **Supplementary Figure 3:** Logistic regression analysis adjusting for prednisone equivalent dose

4. **Supplementary Figure 4:** Interferon (IFN) score during high baricitinib dose period versus low baricitinib dose period.

5. **Supplementary Figure 5:** Proportion of disease flares comparing low baricitinib dose versus high baricitinib dose period when adding IFN score criterion to the flare criteria

I. Supplementary Methods

1. PATIENTS AND INCLUSION AND EXCLUSION CRITERIA

All patients were co-enrolled in the NIH Natural History Protocol of Autoinflammatory Diseases (NCT02974595).

a. **Initial assessment**
   Ten patients with chronic atypical neutrophilic dermatosis with lipodystrophy and elevated temperature/ proteasome-associated autoinflammatory syndrome (CANDLE/PRAAS) treated with baricitinib were included in this retrospective review. Nine out of 10 patients had baricitinib dose reductions and one patient (P8) did not have a dose reduction during the study period (supplementary table 1). Seven patients (P1, P3, P4, P5, P6, P7, P10) had baricitinib dose reduction below the clinically effective dose and two patients (P2 and P9) had minimal dose reduction. Patients with baricitinib dose reductions were included to assess clinical and laboratory symptoms during pre- and post-baricitinib dose reduction periods.
b. **Development of CANDLE/PRAAS Clinical and Subclinical Disease Flare Criteria**
   Post baricitinib dose reduction, five patients (P1, P3, P5, P6, P10) developed clinical symptoms (in addition to laboratory changes) consistent with CANDLE/PRAAS disease flares and two patients (P4 and P7) developed laboratory changes alone that were consistent with disease flare. Data were used from these seven patients to develop CANDLE/PRAAS disease flare criteria.

c. **Assessment of the CANDLE/PRAAS Disease Flare Criteria in visits stratified into high dose and low dose visits**
Six patients (P1, P3, P4, P5, P6, P10) had several visits on low and high baricitinib doses and were included in assessment of the CANDLE/PRAAS flare criteria during visits on high doses and low doses of baricitinib. Patients 2 and 9 were excluded as they did not have a low dose period. Patient 8 was excluded since patient did not have a dose reduction during the study period. Patient 7 was excluded (he was included to development of flare criteria) since patient developed azotemia secondary to presumed BK nephropathy and baricitinib was subsequently discontinued.[1]

2. DAILY DIARY SCORE (DDS) ASSESSMENT

Disease-specific patient daily diary for CANDLE/PRAAS were collected prospectively during JAGA program.[1] Patients with CANDLE/PRAAS or their parents recorded daily symptoms of fever, rash, musculoskeletal pain, headaches, and fatigue. Each symptom was rated on a scale of 0 to 4, with 0=no symptoms, 1=mild symptoms, 2=moderate symptoms, 3=more severe symptoms, and 4=severe symptoms (possible range 0- 20). At each visit, the diary score was calculated as follows:

a. Average score of each symptom was calculated using data entered since the previous visit and correcting for any day for which diary scores were not recorded.
b. The calculated average score for each symptom was summed up and divided by the number of assessed symptoms to calculate the average score for each patient.

Retrospectively reviewed and analyzed DDS data for assessment of baricitinib dose reduction associated clinical flares. Mean DDS was calculated for the period of seven days including the period of three days before dose reduction, day of dose reduction and three days after dose reduction.

If the mean DDS of the reference visit was zero and a patient developed any symptoms during a subsequent visit, it was considered to be a significant change. Therefore, a mean DDS greater than zero is considered indicative of a clinical flare for these patients.

The percent (%) changes in DDS and the laboratory biomarkers were compared between the last visit before dose reduction and the first visit after dose reduction (supplementary table 6a).

3. DATA EXTRACTION

a. **Development of CANDLE/PRAAS Clinical and Subclinical Disease Flare Criteria**

For all visits with dose reductions, clinical and laboratory data were extracted from the last visit prior to baricitinib dose reduction (reference visit), and the first follow-up visit after the dose was reduced (flare visit). Data included DDS, C-reactive protein (CRP), erythrocyte

sedimentation rate (ESR), white blood cell count (WBC), hemoglobin (HGB), absolute lymphocyte count (ALC), platelets (PLT), and 25-gene IFN score[2] (when available). Physician notes were reviewed for documentation of clinical symptoms and physical exam findings. Absolute and percent changes were calculated (supplementary table 6a).

b. Assessment of the CANDLE/PRAAS Disease Flare Criteria in visits stratified into high dose and low dose visits

The visit prior to dose reduction was used as a reference visit to calculate post dose reduction changes. For the assessment of the established disease criteria in high-dose and low-dose visits, we determined a reference visit for each patient separately when the patient was on optimal/clinically effective baricitinib dose and clinically stable considering his/her own disease course, based on expert rheumatologist judgement. For the reference visit, all patients were clinically stable or fulfilled remission criteria that was published by Sanchez et al.[1] Three patients (P4, P5 and P10) were in remission and off glucocorticoid (GC)s. Three patients (P1, P3 and P6) had more than 50% reduction in their daily GC dose from baseline. Patient 7 had reduction in his GC dose from baseline (1.2 mg/kg/day at first JAGA visit) however he was still on 0.82 mg/kg/day GCs at reference visit since he had azotemia and his baricitinib dose was reduced. Laboratory changes after dose reductions that resulted in a clinical flare and laboratory changes after dose reductions that did not result in a clinical flare were systematically evaluated.

For the criteria, DDS increases were used to define clinical symptoms. In a sub-analysis, we assessed the value of adding the IFN score as a laboratory biomarker to increase the sensitivity of disease flare detection and computed the number of additional clinical and subclinical flares that were identified.

4. DEVELOPMENT OF CANDLE/PRAAS DISEASE FLARE CRITERIA

Definition and clarification of data extraction: The number of baricitinib dose reductions, reasons for the dose reductions, the amount of dose reduction, and the first date when the patient took the lower dose were documented. Data were extracted in an excel spreadsheet.

Assessment of daily diary score changes: Clinical notes and daily diaries were retrospectively reviewed for the periods baricitinib dose reductions occurred. Laboratory biomarkers of inflammation and clinical symptoms recorded on a daily diary and/or clinical notes that can constitute a disease flare including fever, rashes, headaches, fatigue, and joint and musculoskeletal pain were extracted. In some instances image of rashes were sent by e-mail. Clinically relevant changes in laboratory biomarkers were included as flare criteria and confirmed previous clinical observations reported in patients with interferonopathies during active disease.[3,4]

Definition of abnormal biomarker cut off values: CRP was considered a clinically abnormal value if 5mg/L or greater, and ESR was considered a clinically abnormal value if 20 mm/hr or

greater. CRP either remained within normal limits or increased to above 5mg/L. Increasing CRP values were a component of the flare criteria. When CRP increased to higher than 5mg/L, the % change was computed. In addition to the change in CRP score resulting in a clinically abnormal value, a cutoff of a 40% increase in CRP was considered a change in cases when the CRP was elevated in the reference visit and a cutoff of a 20% increase in ESR was considered a change in cases when the ESR was elevated in the reference visit. We used the same cut off of 20% and requirement of the change resulting in a clinically abnormal value for IFN score.

Determination of % change in biomarker cut off values: We assessed the laboratory changes at the reference visits and the visits when the patients developed a disease flare post dose reduction (supplementary table 6a) We calculated the % change (increase or decrease) in each laboratory biomarker for each patient comparing the reference visit with the flare visit. Then we determined the lowest meaningful % change for each laboratory biomarker that was associated with clinical symptoms and was consistent with a clinically meaningful change in biomarker. We used these percentages as cut off when developing the flare criteria. We subsequently applied these criteria to all visits to identify possible flare visits during both the low dose and the high dose periods and assessed the % changes to those visits identified as flare visits by using the criteria. The % changes were similar in the high dose and low dose flare visits in the confirmation phase (supplementary table 6b) to those in the baricitinib withdrawal flare visits, which strengthened the notion that the cut off values selected were meaningful in long-term monitoring settings.

5. CONFIRMATION OF THE CANDLE/PRAAS DISEASE FLARE CRITERIA

Definition of a visit: Patients had study visits with clinical and laboratory evaluations, every 3-6 months on average at NIH. However, they were also seen by their local providers and had disease monitoring labs performed in between NIH visits. A patient in a disease flare who requires close monitoring by their local provider may require more frequent clinical and laboratory evaluations. To prevent overinterpretation of data obtained from a period where patients were in states of prolonged disease flare, we determined that each calendar month represents a visit if the patient had clinical and/or laboratory evaluation. If a patient had blood work at multiple occasions within the same calendar month, those multiple visits were considered as one study visit and labeled as flare vs no flare visit based on the worst clinical and laboratory findings. e.g., if a patient had blood work 3 times in a given calendar month and 1 out 3 was consistent with disease flare, we considered this visit as a flare visit.

Definition of high-dose and low-dose visits: To assess the performance of the flare criteria, we assessed the flare criteria during visits when patients received currently recommended dose/clinically effective dose (supplementary table 4),[1 5] and lower than effective dose. Patient visits on lower doses were categorized as “low-dose” visits and those on equal or higher than effective/recommended doses were categorized as “high-dose” visits. In this analysis we only included patients who had both, “low dose” and “high dose” visits (n=6; P1, P3, P4, P5, P6 and P10). We excluded a total of four patients: three patients who had no “low dose” visits (P2, P8 ad P9) and one patient who had azotemia (P7). Patient 7 was excluded since determining the

clinically effective baricitinib dose in the setting of azotemia and renal insufficiency may not be accurate.

We identified 51/153 visits when patients received low dose baricitinib and 102/153 visits when patients received high dose baricitinib, over 9.5 and 25.2 patient years respectively. The same reference visit for each patient was used for comparison with each visit in the low dose and each visit in the high dose period. The clinical and subclinical CANDLE/PRAAS flare criteria were used to calculate the flare rate during low-dose and high-dose visits.

Treatment decisions (steroid adjustments/baricitinib adjustments/no action) during "flare visits": To determine whether clinical and subclinical "flare visits" that were identified by systematic application of the flare criteria had treatment actions implemented in at the time of the visit, we extracted the drug changes and the impact on disease activity on subsequent visits.

BK viral load extraction before and after dose reduction: To assess the impact of the baricitinib dose reduction on viral load in blood and urine, we extracted the viral load at the visit before and after dose reduction and compared whether the load was lower, the same or higher.

IFN score addition as biomarker in a sub analysis:
The 25-gene IFN score[2] was collected as a secondary endpoint in the compassionate use study[1] but was initiated later and was therefore not available for all study visits. Based on control data, a normal IFN score was defined as below 44.2 (cut off is 95%ile in healthy controls).[2]

In the baricitinib reduction visits, the 25-gene IFN score was only available before and after dose reduction for one patient (P1) out of the seven patients who developed a clinical flare post baricitinib dose reduction. The IFN score rose highly when the baricitinib dose was reduced in patient (P1). The original flare criteria were therefore established without the inclusion of the IFN score due to paucity of data. However, for the validation comparing high-dose and low-dose visits, the 25-gene IFN score was available for 108 out of 153 visits, which allowed us to assess the performance of the IFN score at high-dose and at low-dose visits. The median [IQR] IFN score was 45.93 [117.78] in high dose period and 254.46 [267.56] in low dose periods in patients. Difference between median IFN score in low dose versus high dose period was significant ($p<0.0001$) by using two-sided Wilcoxon Rank Sum test.

We then assessed the flare criteria by adding the IFN score to determine whether the flare criteria could capture more "flare visits". The addition of the IFN score to the flare criteria was assessed by determining how many additional flares were identified.

II. Supplementary Tables

Supplementary Table 1 Baricitinib dose reductions and effects on BK viremia/viruria

Patient	# Dose Reduction	Study day<br><br>Pre- and post-reduction baricitinib dose	Dose Reduction by mg (%)	Reason for Dose Reduction	Effect of the baricitinib dose reduction on BK viremia/viruria
P1	#D1*	1342<br><br>from 9 mg/day to 7 mg/day	2 (22)	Intentional: Identify minimal dose that suppresses disease in the context of<br>BK viruria (7.13 log_{10} copy/mL)<br>**BK viremia (low positive <3.7 log_{10} copy/mL)**	BK viruria decreased to 6.6 log_{10} copy/mL<br>***BK viremia resolved***
P2	#D2	1234<br><br>from 7 mg/day to 6 mg/day	1 (14)	Intentional: Identify minimal dose that suppresses disease in the context of<br>BK viruria (3.67 log_{10} copy/mL)<br>No BK viremia	BK viruria decreased to 3.33 log_{10} copy/mL<br>No BK viremia
P3	#D3*	720<br><br>from 10 mg/day to 6 mg/day	4 (40)	Intentional: Anemia (presumed to be baricitinib triggered)<br>BK viruria NA<br>**BK viremia <250 copies/ml**	BK viruria NA<br>***BK viremia resolved***<br>Anemia resolved
	#D4*	1080<br><br>from 8 mg/day to 6 mg/day	2 (25)	Intentional: Identify minimal dose that suppresses disease in the context of<br>BK viruria (>8.7 log_{10} copy/mL)<br>**BK viremia (4.84 log_{10} copy/mL)**	BK viruria decreased to 7.98 log_{10} copy/mL<br>***BK viremia decreased to 3.8 log_{10} copy/mL***
	#D5	2717<br><br>from 8 mg/day to 7 mg/day	1 (12)	Intentional: Identify minimal dose that suppresses disease in the context of<br>BK viruria (>8.6 log_{10} copy/mL)<br>**BK viremia (4.1 log_{10} copy/mL)**	No data on BK viruria data<br>***BK viremia decreased to 3.7 log_{10} copy/mL***
P4	#D6	981<br><br>from 10 mg/day to 4 mg/day	6 (60)	Accidental: Patient ran out of medication and reduced dose to stretch baricitinib until his NIH visit	Not applicable as patient was on the lower dose for only 3 days
	#D7	1061<br><br>from 10 mg/day to 9 mg/day	1 (10)	Intentional: Identify minimal dose maintaining remission in the context of<br>BK viruria (3.56 log_{10} copy/mL)<br>No BK viremia	BK viruria (low viral load prior to dose reduction) remained largely unchanged at relatively low copy numbers, 3.76 log_{10} copy/mL<br>No BK viremia
	#D8	1903<br><br>from 9 mg/day to 8mg/day	1 (11)	Intentional: Increased viral warts<br><br>BK viruria (5.8 log_{10} copy/mL)<br>No BK viremia	BK viruria decreased to 3.8 log_{10} copy/mL<br>No BK viremia
P5	#D9*	1110<br><br>from 11 mg/day to 9 mg/day	2 (18)	Intentional: Identify minimal dose maintaining remission in the context of<br>BK viruria (4.98 log_{10} copy/mL)<br>No BK viremia	BK viruria decreased to 4.12 log_{10} copy/mL<br>No BK viremia
P6	#D10	839<br><br>from 6 mg/day to 4 mg/day	2 (33)	Intentional: Identify minimal dose that suppresses disease in the context of<br>BK viruria (5.8 log_{10} copy/mL)<br>No BK viremia	BK viruria decreased to 4.36 log_{10} copy/mL<br>No BK viremia
P7**	#D11a&	812<br><br>held off for one day	5.4 (100)	Intentional: BK viremia 6.37 log_{10} copy/mL<br>(BK viruria 10.16 log_{10} copy/mL)	see below&

		(from 5.4 mg/day)			
	#D11b[&]	820<br><br>Discontinuation of baricitinib (from 0.9 mg/day)	0.9 (100)	Intentional: Renal failure/Azotemia (presumed BK nephropathy)	Patient continued to have BK viruria $\geq$ 8.9 log_{10} copy/mL and BK viremia $\geq$ 5.99 log_{10} copy/mL in the context of renal failure
P8		No dose reduction during the study period		Not applicable	Not applicable
P9	#D12	216<br><br>from 10 mg/day to 8 mg/day	2 (20)	Intentional: Increase in frequency of headaches	No data on BK viruria and viremia pre-dose reduction
P10[#]	#D13	521<br><br>from 12 mg/day to 10 mg/day	2 (17)	Intentional: Identify minimal dose maintaining remission in the context of<br>BK viruria (9.75 log_{10} copy/mL)<br>No BK viremia	No data on BK viruria (on lower dose of baricitinib)<br><br>No BK viremia
	#D14	899<br><br>from 12 mg/day to 11 mg/day	1 (8)	Intentional: Identify minimal dose maintaining remission in the context of<br>BK viruria (10.28 log_{10} copy/mL)<br>**BK viremia (4.15 log_{10} copy/mL)**[^]	BK viruria >8.6 log_{10} copy/mL<br>***BK viremia decreased to 3.1 log_{10} copy/mL****

D, dose; NA, not available; P, patient

[*]P1: Baricitinib dose was reduced from 9 mg/day to 8 mg/day and then to 7 mg/day four days later, P3: Baricitinib dose was reduced from 10 mg/day to 8 mg/day and then to 6 mg/day the following day. P3: Baricitinib dose was reduced from 8 mg/day to 7 mg/day and then to 6 mg/day 11 days later. P5: Baricitinib dose was reduced from 11 mg/day to 10 mg/day and then to 9 mg about four weeks later.

[**]Patient 7 had one additional dose reduction from 8 mg/day to 6mg/day by patient's local provider at another time point, due to anemia during a hospitalization for a presumed CANDLE/PRAAS disease flare. Baricitinib dose was increased to 8 mg/day 3 weeks later and no laboratory data is available for this dose reduction. This dose reduction was excluded since a. the patient had been admitted with a presumed CANDLE/PRAAS disease flare prior to baricitinib dose reduction, b. missing data for this time frame.

[&]Patient 7's dose reduction started with dose reduction from 8 mg/day to 6 mg/day. Then rapid dose reduction and discontinuation occurred over 3 months in the context of renal failure. Flare occurred with holding baricitinib for one day although it was restarted at a dose of 2.7 mg/day the following day. It was discontinued permanently one week later. After discontinuation of baricitinib, he continued to have active disease that was controlled with high doses of glucocorticoids. He had a major flare presenting as macrophage activation syndrome (MAS). One week after discontinuation of baricitinib, the patient was admitted for persistent fevers, tachycardia, abdominal distention and fluctuating increased work of breathing with increased oxygen requirement.

[#]D13 occurred on study day 521. P10's baricitinib dose was increased from 10 mg/day back to 12 mg/day eventually. On study day 899, P10 had another dose reduction (D14) from 12 mg/day to 11 mg/day.

[^]In addition, patient 10 had mistakenly taken extra dosing (24 mg/day instead of 12 mg/day) for 45 days since he mixed up 4mg vs 1 mg tablets

Baricitinib dose reductions by 1-2 mg/day resulted in lower BK viral load in the blood and urine. BK viremia became negative in P1 and decreased in P3 and P10. With the development of BK nephropathy in one patient (P7), we recommend monitoring BK viremia and suggest keeping BK viral load in blood as low as possible, but all times below log 4 copy/ml (10,000 copies/ml), consistent with recommendations made for kidney transplant recipients.[6,7]

Supplementary Table 2 Summary of post-baricitinib dose reduction clinical symptoms and outcomes of clinical flare vs no clinical flare

Patient/Dose reduction (% Dose change)*		Mean DDS (0-4)	Post Dose Reduction Worsening Clinical Symptoms (Yes/No)	Clinical Flare based on Clinical Judgement (Yes/No)
P1/D#1 (22%)	Pre-dose reduction visit	0.3714	Yes (headaches, oral ulcers, MSK pain, fatigue)	Yes
	Flare Visit	0.428		
	% Change	**15**		
P2/D#2 (14%)	Pre-dose reduction visit	0	No	No
	First visit post dose reduction	NA		
	% Change	NA		
P3/D#3 (40%)	Pre-dose reduction visit	0.13	Yes (fever and rash)	Yes
	Flare Visit	0.4		
	% Change	**208**		
P3/D#4 (25%)	Pre-dose reduction visit	0	Yes (intermittent rash, fever, MSK pain)	No**
	First visit post dose reduction	0.3		
	% Change	**>100**		
P3/D#5 (12%)	Pre-dose reduction visit	0	Yes (intermittent mild rash and MSK symptoms)	No**
	First visit post dose reduction	0.11		
	% Change	**>100**		
P4/D#6 (60%)	Pre-dose reduction visit	0	No	No^
	Flare Visit	0		
	% Change	0		
P4/D#7 (10%)	Pre-dose reduction visit	0.54	No	No
	First visit post dose reduction	0		
	% Change	- >100		
P4/D#8 (11%)	Pre-dose reduction visit	0	No	No
	First visit post dose reduction	0		
	% Change	0		
P5/D#9 (18%)	Pre-dose reduction visit	0.028	Yes (severe headaches and fever)	Yes
	Flare Visit	0.142		
	% Change	**407**		
P6/D#10 (33%)	Pre-dose reduction visit	0.2	Yes (intermittent fevers, fatigue, and rash)	Yes
	Flare Visit	0.31		
	% Change	**55**		
P7/D#11 (100%)	Pre-dose reduction visit	NA	No	No^
	Flare Visit	NA		
	% Change	NA&		
P9/D#12 (20%)	Pre-dose reduction visit	0.114	Yes (fatigue only)	No**
	First visit post dose reduction	0.2		
	% Change	**75**		
P10/D#13 (17%)	Pre-dose reduction visit	0	Yes (periorbital edema with erythema, facial panniculitis, and localized inflammation around thumb)	Yes
	Flare Visit	0.314		
	% Change	**>500**		
P10/D#14 (8%)	Pre-dose reduction visit	0.057	No	No
	First visit post dose reduction	0.028		
	% Change	-50		

Pre-dose reduction visit (=reference visit): Last visit occurred prior to dose reduction.
Flare visit: First visit occurred after baricitinib dose reduction.
*see supplementary table 1 for dose reductions

** Three dose reductions (P3/D#4, P3/D#5, P9/D#12) resulted in changes in DDS and they had labs drawn 2 weeks, 3 months, and 2.5 weeks after recording of the symptoms in DDS respectively. At that time there were no laboratory changes observed. We did not consider these DDS as disease flares.

P3: We observed mild and intermittent rash and MSK symptoms at pre dose reduction visits as well; these symptoms were considered baseline fluctuations. P9: Patient developed fatigue only and DDS change was secondary to fatigue. In the absence of additional CANDLE/PRAAS findings such as fevers, rashes, MSK symptoms, headaches, it was considered to be insufficient to call this as a disease flare. Patient was asymptomatic otherwise.

^P4 received lower dose of baricitinib for three days. Patient had clinically significant laboratory changes post dose reduction with no clinical symptoms. P7's baricitinib was discontinued secondary to azotemia and was on high dose steroids. P7 had clinically significant laboratory changes post dose reduction with no clinical symptoms (both patients fulfilled subclinical flare criteria, please see supplementary table 6a).

D, dose reduction; DDS, daily diary score; NA, not available; P, patient.

Supplementary Table 3 Summary of laboratory biomarkers, mean DDS, glucocorticoid data and clinical status at reference visits for patients included in the confirmation of the flare criteria

Patient*	Mean DDS**	CRP (mg/L)	ESR (mm/hr)	WBC (k/uL)	HGB (g/dL)	PLT (k/uL)	ALC (k/uL)	GC dose (mg/kg/day)	Clinical status
**P1**	0.37142857	0.20	2.00	5.40	14.10	356	1.12	0.3	Stable disease (S)
**P3**	0.13	0.7	40	6.43	7.1^	194	1.52	0.14	Minimal disease activity (MDA)
**P4**	0	3.5	5	7.47	16.6	230	2.47	Off GCs	Remission (REM)
**P5**	0.028	3.3	8	6.42	15	200	3.02	Off GCs	Remission (REM)
**P6**	0.2	0.40	18.00	9.98	12.40	394	0.90	0.33	Stable disease (S)
**P10**	0	1.2	4	4.23	14.2	341	1.2	Off GCs	Remission (REM)

*P7 was not included in the confirmation phase as he was considered to have active disease and remained on high doses of GC.
**Retrospectively reviewed and analyzed DDS data for assessment of baricitinib dose reduction associated clinical flares. Mean DDS was calculated for the period of seven days including the period of three days before dose reduction, day of dose reduction and three days after dose reduction. If the mean DDS of the reference visit was zero and a patient developed any symptoms during a subsequent visit, it was considered to be a significant change. Therefore, a mean DDS greater than zero is considered indicative of a clinical flare for these patients.
^Low hemoglobin was drug related (baricitinib induced anemia)
ALC, absolute lymphocyte count; CRP, C-reactive protein; DDS, daily diary score; ESR, erythrocyte sedimentation rate; GC, glucocorticoid; HGB, hemoglobin; IFN, interferon; P, patient; PLT, platelets; WBC, white blood cell.

At the time of dose reduction, patients P1 and P6 had stable disease (S), P3 had minimal disease activity (MDA), and patients P4, P5, P6 were in clinical remission (REM). See previous definition of remission: DDS<0.15, CRP<5mg/L, off GC. (Sanchez G et al. JCI 2018). We have defined minimal disease activity as: DDS<0.15, CRP<5mg/L and GC less than 0.15mg/kg/day (prednisone equivalent) and stable disease as stable DDS, CRP<5mg/L and stable dose of GC of <0.35mg/kg/day (prednisone equivalent).

Supplementary Table 4 Summary of baricitinib dosing regimen at the time of baricitinib dose reductions

Patient ID	Age at enrollment (years)	Study day reached minimally required optimal dosing	Minimally required optimal dosing (mg/day)[*1]	Study day of dose escalation	Clinically effective baricitinib dose (mg/day)	Study day of baricitinib dose reduction	Baricitinib dose at time of dose reduction (mg/day)
P1	7.3	407	6	820	8	977	7
P3	6.2	127	6	566	8	721	6
P4	19.3	178	8	353	8[**]	982	4
P5	15.8	157	9	164	10	1110	9
P6	2.3	281	4	635	6	839	4
P7	3.5	268	4	396	8	812	0[***]
P10	19.7	148	9	225	11	521	10

[*]Based on Sanchez et al. J Clin Invest. 2018 Jul 2;128(7):3041-3052.
[**]P4 had a dose escalation to 10 mg/day on study day 353 with an attempt to identify the clinically effective dose but later remained stable on 8 mg/day which was determined as clinically effective dose for P4.
[***]P7's dose reduction started with dose decrease from 8 mg/day to 6 mg/day. Then rapid dose reduction and discontinuation occurred over 3 months in the context of renal failure. Flare occurred with holding baricitinib for one day although it was restarted at a dose of 2.7 mg/day the next day. It was discontinued permanently one week later.

Supplementary Table 5 Baseline demographics and clinical characteristics

	Value (%)		Value (%)
**Age at enrollment— yr. mean (min-max)**	11.5 (2.3-19.7)	**DMARDS prior to baseline §– no.**	10 (100)
**Age group — no. (%)**		≥2 DMARDS prior to baseline	7 (70)
0-2 yr	1 (10)	Mean number of DMARDS used prior to baseline (min-max)	3.2 (1-6)
3–6 yr	2 (20)	**Biologics prior to baseline §§– no. (%)**	8 (80)
7–10 yr	2 (20)	≥2 Biologics prior to baseline	7 (70)
11–18 yr	2 (20)	Mean number of biologics used prior to baseline (min-max)	2.8 (0-6)
≥18 yr	3 (30)	**Chronic oral glucocorticoid use §§§ - no. (%)**	8 (80)
**Sex – no. (%)**		Mean exposure to oral glucocorticoids – yr (min-max)	6.7 (1.5-16)
Male	7 (70)	**Clinical manifestations – no. (%)**	
**Race or ethnic group – no. (%)**		Panniculitis-induced lipodystrophy	10 (100)
White	5 (50)	Joint contractures	10 (100)
Black	2 (20)	Myositis‡	8 (80)
Hispanic	3 (30)	Metabolic syndrome*	6 (60)
**By Genetic Diagnosis – no. (%)†**		Systemic inflammation¶	10 (100)
PSMB8	6 (60)	Pulmonary arterial hypertension	1 (10)
PSMB4	1 (10)	Basal ganglia calcifications	7 (70)
PSMB4/PSMB9	2 (20)	Anemia	9 (90)
PSMB8/PSMA3	1 (10)	Lymphopenia	5 (50)
**Autoantibodies**		Height < 3rd percentile	8 (80)
ANA	2(20)	Weight < 3rd percentile	6 (60)
RF	1(10)		
Anti-CCP	0 (0)		

†*PSMB8* (n=5 homozygous, n=1 compound heterozygous), *PSMB4* (n=1 compound heterozygous), *PSMB4/PSMB9* (n=2 digenic), PSMA3/PSMB8 (n=1 digenic)

§ Azathioprine, Colchicine, Cyclosporine, Cyclophosphamide, Dapsone, Hydroxychloroquine, Methotrexate, Mycophenolate mofetil, Tacrolimus, Thalidomide

§§ Adalimumab, Abatacept, Anakinra, Canakinumab, Etanercept, Infliximab, IVIG, Tocilizumab

¶CRP, High Sensitivity >5.0 mg/L or Erythrocyte Sedimentation Rate (ESR) > 20 mm/hr. Patients 6 and 7 had systemic inflammation throughout their disease course however they did not have elevated CRP or ESR at baseline likely because they were on high dose glucocorticoids (> 1 mg/kg/day prednisone equivalent dose) at the time.

‡ Documented by bilateral thigh MRIs

*****By Ford criteria, Ford et al. Diabetes care 2005; 28, 878-81

ANA, antinuclear antibody; anti-CCP, anti–cyclic citrullinated peptide, DMARDS, disease modifying antirheumatic drugs; RF, rheumatoid factor; yr, year.

Supplementary Table 6a Absolute values and percent changes of DDS and laboratory biomarker levels comparing the reference visit with the flare visit

**Patient/ Dose reduction (% Dose change)[*]**		**DDS (0-4)**	**CRP (mg/L)**	**ESR (mm/hr)**	**WBC (k/uL)**	**PLT (k/uL)**	**ALC (k/uL)**	**HGB (k/uL)**	**IFN score (cut off 44.2)**	**Clinical flare (Judgement based)**	**Included to development of CANDLE/ PRAAS flare criteria**	**Fulfilling the CANDLE/PRAAS flare criteria/ Type of flare**
P1/D#1 (22%)	Pre-dose reduction visit	0.3714	0.20	2.00	5.40	356.00	1.12	14.1	31.21	**Yes**	**Yes**	**No**
	Flare Visit	0.428	0.50	5.00	4.88	347.00	0.86	13.6	236.39			**Yes, when including IFN score criterion/ Clinical Flare[^]**
	% Change	**15**	WNL[**]	WNL[**]	-10	-2.5	**-23**	-4	**>500**			
P2/D#2 (14%)	Pre-dose reduction visit	0	4.2	55	5.34	215	1.84	12	11.36	No	No	No
	First visit post dose reduction	NA	0.9	23	5.61	206	2.27	11.9	3.36			
	% Change	NA	WNL[**]	-58	5	-4	23	-1	WNL[**]			
P3/D#3 (40%)	Pre-dose reduction visit	0.13	0.7	40	6.43	194	1.52	7.1	284.5[^^]	**Yes**	**Yes**	**Yes/Clinical Flare**
	Flare Visit	0.4	6.3	70	5.73	130	0.81	6.9	NA			
	% Change	**208**	**>500**	**75**	-11	**-33**	**-47**	-3	NA			
P3/D#4 (25%)	Pre-dose reduction visit	0	3.4	32	5.67	165	1.91	10.1	157.79	No	No	No
	First visit post dose reduction	0.3	1.9	32	5.19	133	2.04	9.2	187.8			
	% Change	**>100**	WNL[**]	0	-8	-19	-7	-9	19			
P3/D#5 (12%)	Pre-dose reduction visit	0	0.6	40	6.14	131	2.33	9.2	166	No	No	No
	First visit post dose reduction	0.11	<5	46	6	131	2	NA	NA			
	% Change	**>100**	WNL[**]	15	-2	0	-14	NA	NA			
P4/D#6 (60%)	Pre-dose reduction visit	0	3.5	5	7.47	230	2.47	16.6	40.19	**No**	**Yes**	**Yes/Subclinical Flare[%]**
	Flare Visit	0	36.9	8	4.7	225	2.09	15.8	35.20			
	% Change	0	**>500**	WNL[**]	**-37**	-2	**-15**	-5	WNL[**]			
P4/D#7 (10%)	Pre-dose reduction visit	0.54	0.7	2	5.74	257	2.26	15.5	8.17	No		No
	First visit post dose reduction	0	3.5	13	7.75	214	2.54	15.9	12.91			
	% Change	->100	WNL[**]	WNL[**]	35	-16	12	3	WNL[**]			
P4/D#8 (11%)	Pre-dose reduction visit	0	2.2	6	5.37	227	2.05	15.1	85.27	No	No	No
	First visit post dose reduction	0	5.5	13	5.6	246	2	15.1	45.93			
	% Change	0	**>100**	WNL[**]	4	8	-2	0	-46			
P5/D#9 (18%)	Pre-dose reduction visit	0.028	3.3	8	6.42	200	3.02	15	-6.96	**Yes**	**Yes**	**Yes/Clinical Flare**
	Flare Visit	0.142	6.3	NA	4.22	153	0.33	13.8	NA			
	% Change	**407**	**91**	NA	**-34**	**-24**	**-89**	-8	NA			
P6/D#10 (33%)	Pre-dose reduction visit	0.2	0.40	18.00	9.98	394.00	0.90	12.4	15.06	**Yes**	**Yes**	**Yes/Clinical Flare**

	Flare Visit	0.31	6.00	25.00	5.92	210.00	1.13	12.9	NA			
	% Change	**55**	**>500**	**39**	**-41**	**-47**	25	4	NA			
P7/D#11 (100%)	Pre-dose reduction visit	NA	0.30	58.00	10.12	135.00	1.47	10.3	-29.38	**No**	**Yes**	**Yes/Subclinical Flare**[%]
	Flare Visit	NA	0.90	NA	6.37	83.00	0.95	8.8	NA			
	% Change	NA	WNL**	NA	**-37**	**-39**	**-35**	-14	NA			
P9/D#12 (20%)	Pre-dose reduction visit	0.114	0.63	7	5.99	280	1.84	13.2	-15	No	No	No
	First visit post dose reduction	0.2	0.4	3	4.49	242	2.03	14.6	-17			
	% Change	**75**	WNL**	WNL**	**-25**	-13	10	10	WNL**			
P10/D#13 (17%)	Pre-dose reduction visit	0	1.2	4	4.23	341	1.2	14.2	102.99	**Yes**	**Yes**	**Yes/Clinical Flare**
	Flare Visit	0.314	27.95	62	2.73	296	0.53	12.6	NA			
	% Change	**>500**	**>500**	**>500**	**-35**	-13	**-56**	-11	NA			
P10/D#14 (8%)	Pre-dose reduction visit	0.057	<0.15	7	3.44	305	0.85	13.9	38.7	No	No	No
	First visit post dose reduction	0.028	0.6	5	4.11	332	0.95	13.8	129.4			
	% Change	-50	WNL**	WNL**	19	9	12	-1	**233**			

Pre-dose reduction visit: Last visit occurred prior to dose reduction. This visit for each patient who developed post dose reduction disease flares was used as a reference visit for evaluation of the flare criteria. Lowest value used to estimate a cutoff value is highlighted in blue. Values that are above the cut off are highlighted in orange.

Flare visit: First visit occurred after baricitinib dose reduction.

*see supplementary table 1 for dose reductions

**within normal limits before and during flare

^Patient fulfilled CANDLE/PRAAS disease flare criteria when including IFN score criterion to the flare criteria. Otherwise, patient was unable to meet required laboratory abnormalities to fulfill the flare criteria although patient developed clinical symptoms in association with baricitinib dose reduction.

^^IFN score not measured for this visit. Used the mean of IFN scores from the visits before and after the reference visit.

%Patient had clinically significant laboratory abnormalities post dose reduction, with no clinical symptoms. Therefore, patient fulfilled subclinical flare criteria.

ALC, absolute lymphocyte count; CANDLE/PRAAS, Chronic atypical neutrophilic dermatosis with lipodystrophy and elevated temperature/proteasome-associated autoinflammatory syndrome; CRP, C-reactive protein; D, dose reduction; DDS, daily diary score; ESR, erythrocyte sedimentation rate; HGB, hemoglobin; IFN, interferon; NA, not available; P, patient; PLT, platelets; WBC, white blood cell; WNL, within normal limits.

Supplementary table 6b Ranges of cut-off values of clinical and subclinical flare visits identified during the high and low dose visit phases are in range with the changes seen in the acute baricitinib withdrawals.

Patient	Max and Min % Change	Mean DDS*	CRP % change (≥40% increase)	ESR % change (≥20% increase)	WBC % change (≥20% decrease)	HGB % change (≥15% decrease)	PLT % change (≥20% decrease)	ALC% change (≥15% decrease)
**"Clinical Flare Visits"**								
P1	Max % change	151	WNL	WNL	-34.8	-19.5	-3.5	-53.6
	Min % change	15.3	WNL	WNL	-20	-1.4	-0.5	-19.6
P3	Max % change	295	2571 (normal to abnormal)	75	-10.9	-2.82	-39.7	-46.7
	Min % change	23	900 (normal to abnormal)	15	-5.4	-2.82	-27.3	-46.7
P5	Max % change	1935.7	163.6 (normal to abnormal)	WNL	-40.8	-8	-29.5	-89
	Min % change	103.57	90.9 (normal to abnormal)	WNL	-11.8	-2	-11	-45.3
P6	Max % change	230	1400 (normal to abnormal)	38.9 (normal to abnormal)	-42.6	-15.3	-58.9	not applicable*
	Min % change	15	WNL	WNL	-29.9	-5.6	-33.5	not applicable*
P10	Max % change	Increased from zero, unable to calculate	2229 (normal to abnormal)	1450 (normal to abnormal)	-35.4	-15.5	-23.5	-69.2
	Min % change	Increased from zero, unable to calculate	665.8 (normal to abnormal)	900 (normal to abnormal)	-3.1	-9.9	-12.3	-24.2
**"Subclinical Flare Visits"****								
P1	Max % change	not applicable	WNL	WNL	-32.78	-15.60	not applicable*	-22.32
	Min % change	not applicable	WNL	WNL	-32.78	-15.60	not applicable*	-22.32
P3	Max % change	not applicable	2100 (normal to abnormal)	192.5	-22.71	not applicable*	-41.75	-18.42
	Min % change	not applicable	1142.8 (normal to abnormal)	22.5	-22.71	not applicable*	-23.71	-2.63
P4	Max % change	not applicable	954.29 (normal to abnormal)	300 (normal to abnormal)	-37.08	-16.27	-2.17	-19.03
	Min % change	not applicable	48.57 (normal to abnormal)	WNL	-25.03	-9.04	-2.17	-15.38

P6	Max % change	not applicable	4400 (normal to abnormal)	233.3 (normal to abnormal)	-51.80	-8.06	-56.09	not applicable*
	Min % change	not applicable	WNL	WNL	-36.37	-4.84	-50.25	not applicable*
**OVERALL**	**Max % change**	**1935.7**	**4400**	**1450**	**-51.8**	**-19.5**	**-58.9**	**-89**
**OVERALL**	**Min % change**	**15**	**48.57**	**15**	**-3.1**	**-1.4**	**-0.5**	**-15.38**

The ranges of percent changes of components of the flare criteria were extracted for each patient are summarized in the table. The lowest value that is making the cut off is highlighted in blue. All other values fulfilling the flare criterion are highlighted in orange.

The lowest value that is making the cut off is highlighted in blue. All other values fulfilling the flare criterion are highlighted in orange.

*increased

**DDS change not applicable as patients had <15% during subclinical flare visits by definition

ALC, absolute lymphocyte count; CRP, C-reactive protein; D, dose reduction; DDS, daily diary score; ESR, erythrocyte sedimentation rate; HGB, hemoglobin; IFN, interferon; Max, maximum; Min, minimum; P, patient; PLT, platelets; WBC, white blood cell; WNL, within normal limits

Supplementary Table 7 Treatment decisions (steroid adjustments/baricitinib adjustments/no action) during "flare visits"

Patient	Subclinical /Clinical Flare*	Presumed cause of flare**	GC treatment action (yes/no)	Baricitinib treatment action (yes/no)***	Outcome
**Post baricitinib dose reduction visits**					
P1	Clinical	Intentional baricitinib dose reduction from 9 mg/day to 7 mg/day<br>Ind: Find lowest tolerated dose<br>RV: Stable	Yes, increased from 0.3 mg/kg/day to 0.43 mg/kg/day	No, remained on baricitinib 7 mg/day	Resolution of flare with GC increase<br><br>**Flare criteria fulfilled with IFN score only → increase GC dose, temporary resolution of flare**<br><br>**Flare recurred with GC dose decrease.**
P3	Clinical	Intentional baricitinib dose reduction from 8 mg/day to 6 mg/day<br>Ind: Manage side effect, anemia<br>RV: MDA	Yes, GC increased from 4 mg/day [0.14 mg/kg/day] to 6 mg/day [0.21 mg/kg/day] 6 weeks after baricitinib dose reduction	Yes, increased from 6 mg/day to 8 mg/day 3 months after baricitinib dose reduction	Resolution of Flare, GCs tapering<br><br>**Flare criteria fulfilled → adjust baricitinib dose back to baseline**<br><br>**Ability to lower CG on baricitinib 8mg/day**
P4	Subclinical	Accidental baricitinib dose reduction from 10 mg/day to 4mg/day<br>RV: REM	No, off GCs	Yes, increased from 4 mg/day to 10 mg/day 3 days after	**Restart higher dose of baricitinib**<br>"Resolution of Flare, off GCs"<br><br>**Flare criteria fulfilled → adjust baricitinib dose back to baseline, patient remains off GCs**
P5	Clinical	Intentional baricitinib dose reduction from 10 mg/day to 9 mg/day<br>Ind: Find lowest tolerated dose<br>RV: REM	No, off GCs	Yes, increased from 9 mg/day to 10 mg/day one week after	Resolution of Flare, off GCs<br><br>**Flare criteria fulfilled → adjust baricitinib dose back to baseline, patient remains off GCs**
P10	Clinical	Intentional baricitinib dose reduction from 12mg/day to 10mg/day<br>Ind: Find lowest tolerated dose<br>RV: REM	Yes, was off GCs and required 2 short courses of GCs one month and two months after dose reduction	Yes, increased from 10 mg/day to 12 mg/day one month after dose reduction	Resolution of Flare after baricitinib and GC increase, later able to wean off GCs!<br><br>**Flare criteria fulfilled → adjust baricitinib dose and GC dose then able to wean GC**
P6	Clinical	Intentional baricitinib dose reduction from 6mg/day to 4 mg/day<br>Ind: Keep viral load low<br>RV: Stable	No, remained on GCs 3 mg/day [0.16 mg/kg/day]	No, remained on baricitinib 4 mg/day	**Persistent flare till end of study. Had 8/14 (57%) flare visits over ~2.5 yrs**<br><br>**Flare criteria fulfilled → no baricitinib and no GC dose adjustment → cont' flare (see below)**

**“Flare visits” in low dose period (n=17)** ***identified in validation phase of "flare criteria”***					
P1	Clinical	GC taper to 0.3 mg/kg/day	No	No, remained on baricitinib 7 mg/day	**Had 3/12 (25%) flare visits over 2 years**.<br><br>**Flare criteria fulfilled → no baricitinib dose and no GC dose adjustment → 25% flare on GC 0.3mg/kg/d**
	Clinical	NA	No		
	Clinical	NA<br><br>Ind: Lower steroid dose<br>RV: Stable	No		
P3	Clinical	NA	No, remained on 3.5 mg/day [0.08-0.10 mg/kg/day].	No, remained on 6 mg/day	**Had 5/13 (38%) flare visits over 2.5 years.**<br><br>**Flare criteria fulfilled → no adjustment of baricitinib or GC dose → 38% flare rate on GC 0.1mg/kg/d**
	Clinical	NA			
	Subclinical	NA			
	Subclinical	NA			
	Subclinical	NA<br><br>Ind: Manage side effect, anemia<br>RV: MDA)			
	Clinical	GC taper to 0.06 mg/kg/day	No	No, remained on baricitinib 7 mg/day	**Had 2/6 (33%) flare visits over 13 months.**<br><br>**Flare criteria fulfilled → higher baricitinib dose (7mg/d) allowed further GC reduction to 0.06mg/kg/day.**
	Subclinical	NA<br><br>Ind: Manage side effect, anemia<br>RV: MDA)	No, remained on 3 mg/day [0.06 mg/kg/day]		
P6	Subclinical	NA	No, remained on 2.5-3 mg/day [0.10-0.16 mg/kg/day]	No, remained on baricitinib 4 mg/day	**Had 8/14 (57%) flare visits over ~2.5 yrs**<br><br>**Flare criteria fulfilled → baricitinib dose (4 mg/d and GC 0.16mg/kg/d with continued intermittent flares**
	Clinical	NA			
	Clinical	NA			
	Subclinical	NA			
	Clinical	NA			
	Subclinical	NA			
	Subclinical	NA<br><br>Ind: Keep viral load low Parental worries.<br>RV: Stable			
**“Flare visits” in high dose period (n=12)** ***identified in validation phase of "flare criteria”***					
P1	Clinical	GC taper to 0.35 mg/kg/day	Yes, increased from 0.35 mg/kg/day to 0.38 mg/kg/day	No, remained on baricitinib 8 mg/day	Had 2/3 (66%) flare visits, on GCs at ~0.35 mg/kg/day over 10 months<br><br>**Flare criteria fulfilled → no baricitinib and no GC change →ongoing disease activity**
	Clinical	GCs tapering to 0.3mg/kg/day<br><br>Ind: Lower steroid dose<br>RV: Stable	No	**Yes, increased to 9 mg/day**	**Had 0/3 (0%) flare visits, GCs tapering to 0.3 mg/kg/day over 8 months**<br><br>**Flare criteria fulfilled → higher baricitinib dose (9mg/d) GC 0.3 mg/kg/day optimally protected the patient.**
	Clinical	NA	No	No, remained on baricitinib 8 mg/day	Had 3/8 (38%,) flare visits over 2 years and was able to taper GC to 0.14 mg/kg/day.<br><br>**Flare criteria fulfilled → baricitinib dose (8mg/d) allowed further GC reduction to 0.14 mg/kg/day but with overall poorer control than at the 9 mg/day dose.**
	Subclinical	NA	No		
	Clinical	GC taper:6.5 mg/day 0.23 mg/ kg/day to 5 mg/day 0.13 mg/ kg/day]<br><br>Ind: Lower steroid dose	No		

		RV: Stable			
	Clinical	NA	**No, remained on ~0.13 mg/kg/day**	**No, remained on 9 mg/day**	Had 1/4 (25%) over 9 months, on GCs 0.14mg/kg/day, no change from prior visits<br><br>**Flare criteria fulfilled → higher baricitinib dose (9mg/d) on GC dose 0.14 mg/kg/day.**
P4	Subclinical	NA	No, remained on baricitinib 8mg/day	Had 2/21 (9%) flare visits over 5 years, off GCs<br><br>(*adolescent-adult transition*)	Had 2/21 (9%) flare visits over 5 years.<br><br>(*adolescent-adult transition*)<br><br>**Flare criteria fulfilled → with baricitinib taper to (8mg/d), no GC.**
	Subclinical	NA<br><br>Ind: Find lowest tolerated dose<br>RV: REM			
P5	Clinical	NA	No, off GCs	No, remained on baricitinib 10 mg/day	Had 3/17 (18%) flare visits over 4.5 years, off GCs
	Clinical	NA			
	Clinical	NA<br>Ind: Find lowest tolerated dose<br>RV: REM			
P10	Clinical	NA	No, off GCs	No, remained on baricitinib 12 mg/day	Had 2/11 (18%) flare visits off GCs<br><br>(*adolescent-adult transition*)
	Clinical	NA<br>Ind: Find lowest tolerated dose<br>RV: REM	No, off GCs		
**Additional high-dose Visits**					
P3	Patient had 12 high-dose visits over ~2.5 years and did not have clinical or subclinical flare. During these high-dose visits, patient was on baricitinib 10 mg/day for 3 months and on 8 mg/day for ~2.2 years. Overall, achieved GC tapering down to 0.09 mg/kg/day				Flare rate 0%, GCs tapering
P6	Patient had 3 high-dose (on baricitinib 6 mg/day) visits over 6.8 months and did not have clinical or subclinical flare. Achieved GC tapering from 0.46 mg/kg/day to 0.16 mg/kg/day				Flare rate 0%, GC tapering

*all visits that were identified by fulfilling the proposed flare criteria (clinical and subclinical flares) in the validation phase are listed here.
** in column presumed cause of flare, we added the reason for the presumed flare i.e glucocorticoid taper, baricitinib dose reduction, other. We also determined the level of disease control the patient had prior to the flare at the reference visit (RV): remission (REM) meaning DDS<0.15, normal CRP and off steroids (P4, P5, P10) , minimal disease activity (MDA), DDS<0.15, normal CRP, prednisone equivalent <0.15mg/kg/day (P3) and stable for patient who normalized CRP but had still elevated DDS and were on higher doses of steroids (P1, P6, P7). See supplementary table 7. P7 was on high doses of steroids 0.8 mg/kg/day and could not be tapered. P7 was not included in the validation of the criteria.
***all baricitinib dose increases were within the dose range of the provided dosing table (Kim at al. 2018)
GC, glucocorticoid; Ind, indication; MDA: minimal disease activity; NA, not available; P, patient; REM, remission; RV, reference visit.

<u>Actual clinical scenarios observed:</u>

Scenario 1: In patient P4 who had subclinical flares only and in patients P5 and P10 who had clinical flares after fulfilling remission criteria off GC, the baricitinib dose was adjusted after a baricitinib dose reduction during "low-dose visits". The flare resolved in P4 and P5 by adjusting the baricitinib dose back to baseline. In P10, two short courses of GCs were required in addition to a baricitinib dose adjustment to achieve remission again.

However, flares identified in the validation period during the "high dose visits" did not result in GC or baricitinib changes. On subsequent visits P4, P5 and P10 fulfilled flare criteria on 9%, 18% and 18% of visits respectively.

Scenario 2: Post flare P1 (S, stable disease) remained on baricitinib 7 mg/day and GC doses between 0.23-0.30 mg/kg/day. Over the next ~2 years he had 3 clinical flare visits out of 12 low-dose visits (25% flare visits) and was unable to wean GC. The baricitinib dose was adjusted to 8 mg/day and GCs could be tapered to 0.14 mg/kg/day, however he had 2 clinical and 1 subclinical flare visits out of 8 high-dose visits (38% flare visits) over 2 years. Eventually the baricitinib dose was increased to 9 mg/day, and he had 1 clinical flare visit out of 4 high-dose visits (25% flare visits) and remained on GCs 0.14 mg/kg/day.

In patients P3 (MDA) and P6 (S, stable disease) the baricitinib dose reduction without subsequent dose adjustment resulted in 38% and 57% of subsequent visits fulfilling "flare criteria" respectively during a period of ~2.5 years. In P3 the baricitinib dose was eventually increased to 8 mg/day which allowed a GC taper to doses below 0.15mg/kg/day (criteria for MDA) with no subsequent clinical or subclinical flares (flare rate 0%). In P6 the family elected to stay on a lower baricitinib dose and not adjust baricitinib or GC dose.

Suggestion: The proposed flare criteria can be used to manage patients with CANDLE/PRAAS in remission or with MDA. The scenarios above illustrate their use in finetuning treatment and adjusting steroid doses to the lowest dose possible in patients with MDA. In patients who achieved remission, the "flare rates" may help in quantifying disease control long-term and to better characterize the "level of disease control" that can be achieved on treatment with janus kinase inhibitors.

Proposed use of criteria for monitoring CANDLE/PRAAS patients who are in clinical remission (P4, P5, P10) or have minimal disease activity (P3), clinical scenarios:

1. Baricitinib dose reductions to determine the lowest dose tolerated with the goal to:
   a. Keep BK viral load in blood as low as possible but below log 4 copy/ml (10,000 copies/ml) at all times, consistent with recommendations for BK viral load monitoring for kidney transplant recipients. [6 7] **P3** (MDA)**)**
   b. Manage side effects such as anemia **(P3)** (MDA).
   c. Find lowest tolerated dose in patients in remission or with minimal disease activity **(P3** (MDA) **P4** (REM), **P5** (REM), **P10** (REM)**)**

2. Lower steroids doses in patients with MDA

The use of the criteria in patients who <u>are not</u> in clinical remission or fulfill criteria for MDA, but have stable disease needs to be evaluated prospectively in a larger cohort. Clinical scenarios (P1, P6) where they may be useful include:

1. Baricitinib dose reductions to:
   a. Keep BK viral load in blood as low as possible but below log 4 copy/ml (10,000 copies/ml) at all times, consistent with recommendations for BK load monitoring for kidney transplant recipients. [6 7] (**P1** (S), **P6** (S)**)**
   b. Manage side effects such as anemia.
2. Lower steroid doses in patients on GC doses, that are too high to achieve catch up growth (>0.15mg/kg/day) (**P1** (S), **P6** (S)**)**

The flare criteria are not useful for patients who have not achieved disease control.
P7 (active, high dose of prednisone, excluded) never achieved disease control. Baricitinib was withdrawn due to BK nephrotoxicity.

III. Supplementary Figures

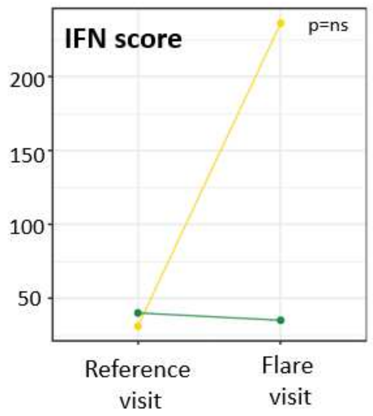

Supplementary Figure 1: Acute IFN score change with baricitinib dose reduction. This figure depicts comparison of the reference visit (=last visit before baricitinib dose reduction) with flare visit (=the first visit after dose reduction) for IFN score in patient 1 (yellow line) and patient 4 (green line). Patient 4 achieved remission nine months prior to baricitinib dose reduction and was in long term remission at the time of dose reduction. A two-sided nonparametric Wilcoxon signed rank test with uncorrected p-values were used to underscore the descriptive representation. IFN, interferon.

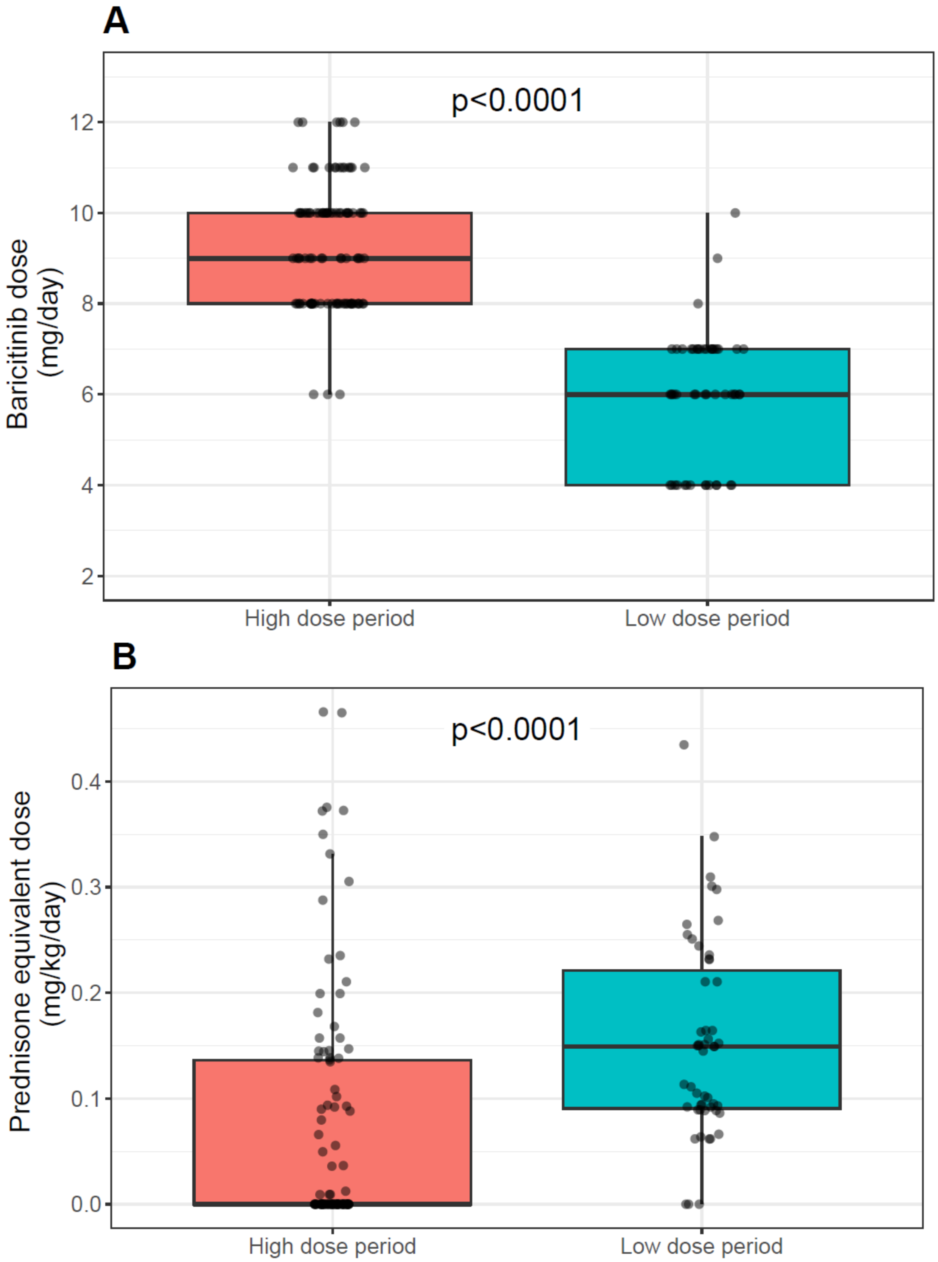

Supplementary Figure 2: Baricitinib and prednisone equivalent dose high baricitinib dose period versus low baricitinib dose period. A. The baricitinib dose was significantly higher in the high dose period compared to low dose period (median [IQR]: 9.00 [2.00] mg/day and 6.00 [0] mg/day respectively, p<0.0001). B. The prednisone equivalent dose was significantly lower in the high dose period compared to the low dose period (median [IQR]: 0.00 [0.136] mg/kg/day and 0.149 [0.13] mg/kg/day respectively, p<0.0001)

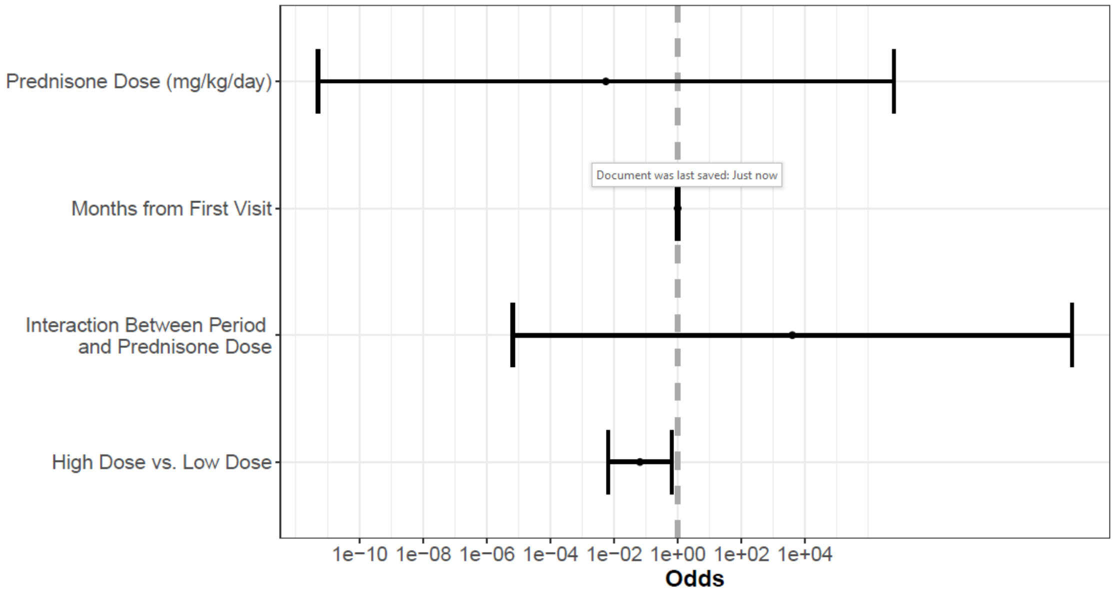

Supplementary Figure 3: Logistic regression analysis adjusting for prednisone equivalent dose. A total of 153 visits were assessed using generalized estimating equations with a correction for small sample (saws citation) given multiple visits are present per participant. Adjusting for prednisone equivalent dose, higher odds of a flare in the low baricitinib dose period than in the high baricitinib dose period are found (p=0.032). There are no significant associations with the months from first visit and prednisone. An interaction term between period (low vs high dose period) and mean prednisone dose to determine if the association between period and flare depends on the level of prednisone dose. There is not significant interaction between period and prednisone dose.

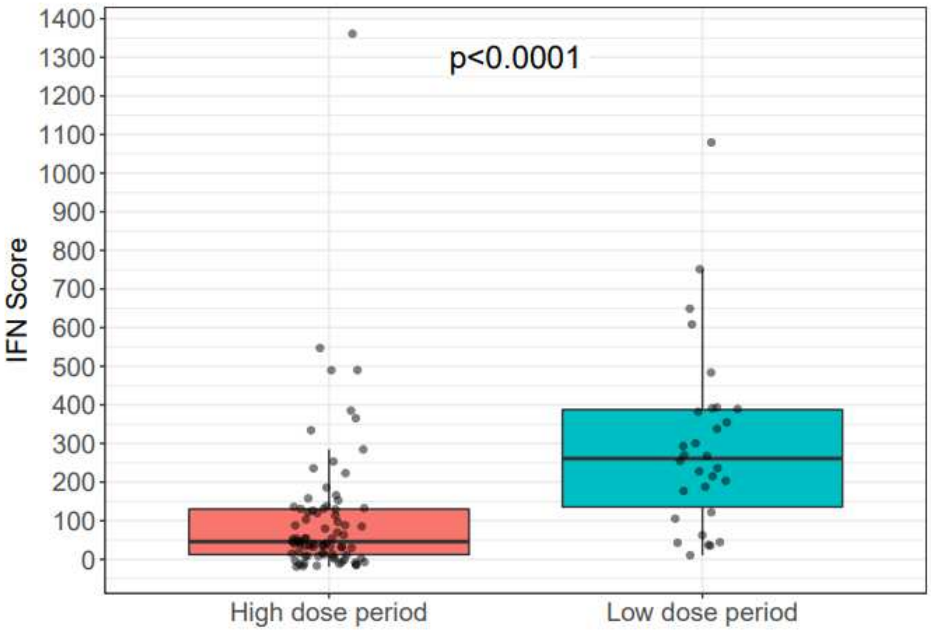

Supplementary Figure 4: Interferon (IFN) score during high baricitinib dose period versus low baricitinib dose period. A total of 153 visits from six patients were identified during the low dose and high dose baricitinib periods. Of 153 visits, the IFN score was measured in 108 visits (n=29 low dose visits and n=79 high dose visits). The median [IQR] IFN score was 45.93 [117.78] in the high dose period and 254.46 [267.56] in low dose period. Difference between median IFN score in low dose versus high dose period is significant ($p<0.0001$). A two-sided Wilcoxon Rank Sum test was used for comparison.

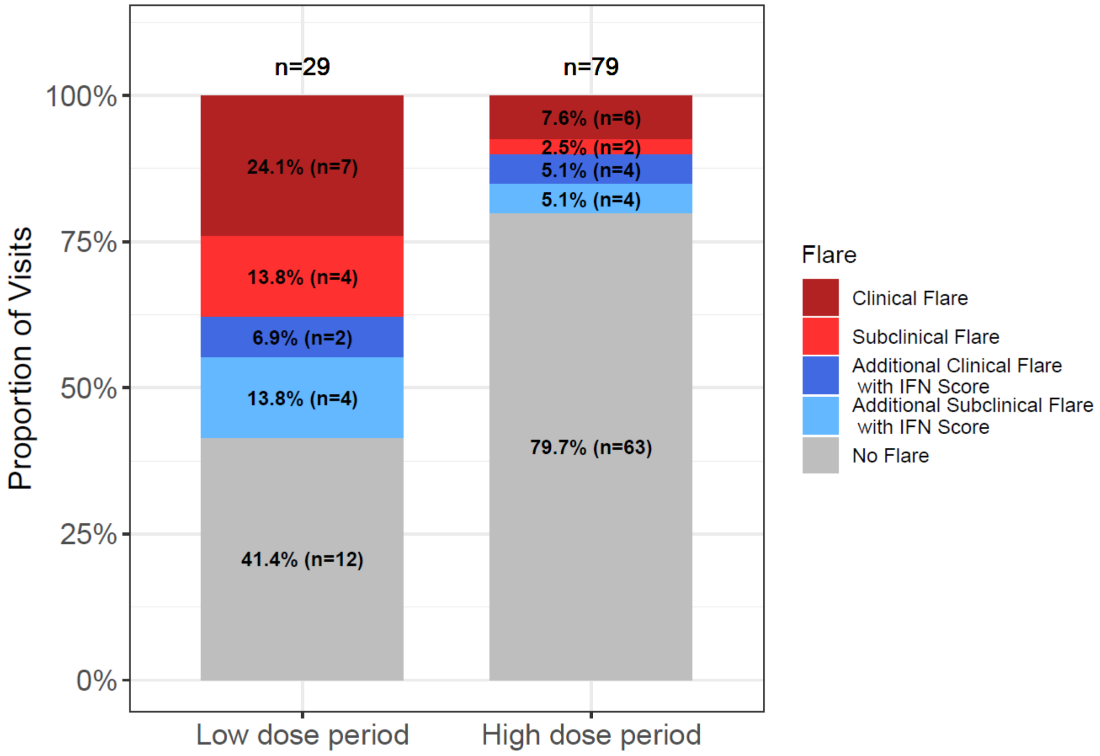

Supplementary Figure 5: Comparison of proportion of disease flares in baricitinib low dose (n=29) and high dose (n=79) visits when adding IFN score criterion to the flare criteria. This sub analysis compares the flare rate when using the flare criteria without the IFN score (red) and with the IFN score (blue). A total of 108 visits with available IFN score could be included in the analysis, of these 79/108 visits and low dose period consists of 29/108 visits. The proportion of visits when patients fulfilled flare criteria during the low dose and high dose period was calculated separately for flare criteria without and with the IFN score using a two-sided chi-squared test of homogeneity. Proportion of visits that patients fulfilled the flare criteria during the low dose period (37.93%, n=11/29) is significantly higher (p<0.001) than during the high dose period (10.13%, n=8/79). When adding the IFN score criterion to the flare criteria, we identified six additional clinical flares and eight additional subclinical flares, four in low-dose visits and four in the high-dose visits. The proportion of visits that patients fulfill flare criteria increased from 37.93% to 58.62% (n=17/29) during the low dose period and from 10.13% to 20.25% (n=16/79) during the high dose period. The difference is significant (p<0.001).

Supplementary References

1. Sanchez GAM, Reinhardt A, Ramsey S, et al. JAK1/2 inhibition with baricitinib in the treatment of autoinflammatory interferonopathies. *J Clin Invest* 2018;128(7):3041-52. doi: 10.1172/JCI98814 [published Online First: 20180611]
2. Kim H, de Jesus AA, Brooks SR, et al. Development of a Validated Interferon Score Using NanoString Technology. *J Interferon Cytokine Res* 2018;38(4):171-85. doi: 10.1089/jir.2017.0127
3. Canna SW, Goldbach-Mansky R. New monogenic autoinflammatory diseases--a clinical overview. *Semin Immunopathol* 2015;37(4):387-94. doi: 10.1007/s00281-015-0493-5 [published Online First: 20150512]
4. Lin B, Goldbach-Mansky R. Pathogenic insights from genetic causes of autoinflammatory inflammasomopathies and interferonopathies. *J Allergy Clin Immunol* 2022;149(3):819-32. doi: 10.1016/j.jaci.2021.10.027 [published Online First: 20211208]
5. Kim H, Brooks KM, Tang CC, et al. Pharmacokinetics, Pharmacodynamics, and Proposed Dosing of the Oral JAK1 and JAK2 Inhibitor Baricitinib in Pediatric and Young Adult CANDLE and SAVI Patients. *Clin Pharmacol Ther* 2018;104(2):364-73. doi: 10.1002/cpt.936 [published Online First: 20171208]
6. Kasiske BL, Zeier MG, Chapman JR, et al. KDIGO clinical practice guideline for the care of kidney transplant recipients: a summary. *Kidney Int* 2010;77(4):299-311. doi: 10.1038/ki.2009.377 [published Online First: 20091021]
7. Hirsch HH, Randhawa P, Practice ASTIDCo. BK virus in solid organ transplant recipients. *Am J Transplant* 2009;9 Suppl 4:S136-46. doi: 10.1111/j.1600-6143.2009.02904.x
